# Use of In Vivo Imaging and Physiologically-Based Kinetic Modelling to Predict Hepatic Transporter Mediated Drug–Drug Interactions in Rats

**DOI:** 10.3390/pharmaceutics15030896

**Published:** 2023-03-10

**Authors:** Nicola Melillo, Daniel Scotcher, J. Gerry Kenna, Claudia Green, Catherine D. G. Hines, Iina Laitinen, Paul D. Hockings, Kayode Ogungbenro, Ebony R. Gunwhy, Steven Sourbron, John C. Waterton, Gunnar Schuetz, Aleksandra Galetin

**Affiliations:** 1 Centre for Applied Pharmacokinetic Research, Division of Pharmacy and Optometry, School of Health Science, The University of Manchester, Manchester M13 9PL, UKdaniel.scotcher@manchester.ac.uk (D.S.);; 2 SystemsForecastingUK Ltd., Lancaster LA1 5DD, UK; 3 Bioxydyn Ltd., Manchester M15 6SZ, UK; 4 MR & CT Contrast Media Research, Bayer AG, 13353 Berlin, Germany; 5 GSK, Collegeville, PA 19426, USA; 6 Sanofi-Aventis Deutschland GmbH, Bioimaging Germany, 65929 Frankfurt am Main, Germany; 7 Antaros Medical, 431 83 Mölndal, Sweden; 8 MedTech West, Chalmers University of Technology, 413 45 Gothenburg, Sweden; 9 Department of Infection, Immunity and Cardiovascular Disease, University of Sheffield, Sheffield S10 2TA, UK; 10 Centre for Imaging Sciences, Division of Informatics Imaging & Data Sciences, School of Health Sciences, The University of Manchester, Manchester M13 9PL, UK

**Keywords:** gadoxetate, pharmacokinetics, hepatic transporters, modelling and simulation, DCE-MRI, OATP1B

## Abstract

Gadoxetate, a magnetic resonance imaging (MRI) contrast agent, is a substrate of organic-anion-transporting polypeptide 1B1 and multidrug resistance-associated protein 2. Six drugs, with varying degrees of transporter inhibition, were used to assess gadoxetate dynamic contrast enhanced MRI biomarkers for transporter inhibition in rats. Prospective prediction of changes in gadoxetate systemic and liver AUC (AUCR), resulting from transporter modulation, were performed by physiologically-based pharmacokinetic (PBPK) modelling. A tracer-kinetic model was used to estimate rate constants for hepatic uptake (k_he_), and biliary excretion (k_bh_). The observed median fold-decreases in gadoxetate liver AUC were 3.8- and 1.5-fold for ciclosporin and rifampicin, respectively. Ketoconazole unexpectedly decreased systemic and liver gadoxetate AUCs; the remaining drugs investigated (asunaprevir, bosentan, and pioglitazone) caused marginal changes. Ciclosporin decreased gadoxetate k_he_ and k_bh_ by 3.78 and 0.09 mL/min/mL, while decreases for rifampicin were 7.20 and 0.07 mL/min/mL, respectively. The relative decrease in k_he_ (e.g., 96% for ciclosporin) was similar to PBPK-predicted inhibition of uptake (97–98%). PBPK modelling correctly predicted changes in gadoxetate systemic AUCR, whereas underprediction of decreases in liver AUCs was evident. The current study illustrates the modelling framework and integration of liver imaging data, PBPK, and tracer-kinetic models for prospective quantification of hepatic transporter-mediated DDI in humans.

## 1. Introduction

Clinically relevant drug–drug interactions (DDIs) can result in potentiated or reduced efficacy, that requires drug dose adjustment. In addition, DDIs can potentially increase or reduce drug toxicity to liver or other tissues, and may arise via alterations in activities of transport proteins that mediate uptake into hepatocytes and/or biliary excretion of drugs. For example, inhibition of the hepatic uptake transporter organic anion transporting polypeptide 1B1 (OATP1B1), by a co-administered perpetrator drug, will affect hepatic clearance of many statins and lead to elevated plasma and systemic tissue exposure, thereby causing myotoxicity [1,2]. Conversely, interaction of perpetrator drugs with hepatic transporters that mediate biliary excretion, may alter hepatocyte exposure to a victim drug without causing a measurable effect on systemic plasma exposure (e.g., metformin DDIs due to organic cation transporter 2 (OCT2) and multidrug and toxin extrusion protein (MATE)1 and MATE2-K inhibition [3]).

Quantitative translation of in vitro data through in vitro–in vivo extrapolation (IVIVE), can be undertaken via physiologically-based pharmacokinetic (PBPK) modelling, which integrates in vitro transporter kinetic/inhibition data with relevant physiological parameters [4,5]. PBPK models simulate changes in both systemic and tissue exposure of the victim drug, that arise because of changes in enzyme and/or transporter activity caused by the perpetrator drug [6,7]. These methods are used routinely to support regulatory submissions and drug labelling, and their value has been recognized in DDI and PBPK regulatory guidance documents [8,9,10]. However, verification of the accuracy of PBPK simulations of tissue exposure is challenging, especially for transporter DDIs where changes in drug exposure may occur within hepatocytes, but not in plasma (or not to the same extent). In particular, clinical DDI studies are usually unable to detect interactions that arise via inhibition of hepatobiliary efflux transporters, such as multidrug resistance-associated protein (MRP)2, due to the lack of a measurable effect on systemic plasma exposure [6].

Hence there is a need for additional methods to quantify effects of test drugs on transporter function in vivo. One promising approach is dynamic contrast-enhanced magnetic resonance imaging (DCE-MRI), using the contrast agent gadoxetate. Since magnetic resonance imaging (MRI) is tomographic, concentrations of gadoxetate can be simultaneously determined in plasma, liver, kidney, bile, and other compartments. Gadoxetate is administered intravenously (iv) and is eliminated exclusively via renal and biliary excretion. It is a substrate of multiple hepatocyte uptake (OATP1A1, OATP1B1, OATP1B3, and Na^+^-taurocholate cotransporting polypeptide (NTCP)) and efflux (MRP2, MRP3) transporters [11,12,13], and has a high hepatic extraction ratio in rats. Uptake of gadoxetate in healthy hepatocytes, enhances the regional T_1_-weighted magnetic resonance (MR) signal. The opposite is seen in the presence of lesions from liver metastases (non-hepatic origin). These properties have been exploited in clinical MRI, as gadoxetate is used routinely to detect and characterize lesions in adults with known or suspected focal liver disease [14].

If MR is monitored dynamically during the uptake and washout of the contrast agent, then regional gadoxetate pharmacokinetics can be derived, to probe hepatic transporter-mediated DDI in human. Data from such DCE-MRI experiments are often analyzed using multi-compartment tracer-kinetic models [6,15,16,17,18,19,20]. (An indicator is a detectable substance that is introduced into a physiological system, yielding information about the system itself. A tracer is a type of indicator chemically identical to a substance of interest but separately detectable. Gadoxetate is an indicator, but not a tracer. However, it is a common convention to refer to tracer-kinetic models as models for indicators that are not tracers, such as gadoxetate [15].) These tracer-kinetic models, unlike PBPK models, do not assess the mass balance in the whole body. Tracer-kinetic models use the systemic concentration (e.g., in arterial and/or portal venous blood vessels) as input function to a compartmental model describing only the organs or tissues of interest (e.g., the liver) [15].

PBPK models have also been applied to DCE-MRI data, although not as commonly as tracer-kinetic models [21,22]. We previously reported a PBPK model for gadoxetate in rats, developed using a combination of bottom-up (i.e., based on IVIVE) and top-down approaches. The PBPK model captured the blood, spleen, and liver gadoxetate DCE-MRI profiles of both control and inhibitory phases, following the administration of an intravenous rifampicin dose of 10 mg/kg bodyweight [23]. With the same DCE-MRI data, we also developed a compartmental tracer-kinetic model giving the kinetic rate constants for gadoxetate transport from the extracellular space into hepatocytes (k_he_), and from hepatocytes into bile (k_bh_). The reproducibility of the assay, and the effect of rifampicin 10 mg/kg on k_he_ and k_bh_, were also assessed [24]. The results of this previous work showed promising use of gadoxetate as an imaging probe to investigate the effects of perpetrator drugs on hepatic transporters in rats [23,24].

The aim of the present study was to further evaluate the imaging biomarker gadoxetate, for investigation of hepatic transporter mediated DDI, using DCE-MRI data. To that end, two modelling approaches, PBPK and tracer-kinetic models, were investigated, using gadoxetate–drug interaction data obtained with six test drugs in rats. The drugs were selected for having variable in vitro potency of OATP1B inhibition and/or drug-labeling for potential to cause drug-induced liver injury (DILI), namely ciclosporin, rifampicin, bosentan, ketoconazole, asunaprevir, and pioglitazone. Appropriate doses for all the six drugs were selected by pharmacokinetic modelling and simulation. The translational modelling capabilities of the previously developed gadoxetate PBPK model [23], were assessed by comparing the prospective bottom-up prediction of the hepatic transporters DDI, with the observed gadoxetate DCE-MRI systemic and liver data in the inhibitory phases. In parallel, a tracer-kinetic model [24] was evaluated by quantifying the effect of the six drugs investigated, on gadoxetate hepatic volume transfer constant (K^trans^), and rate constants k_he_ and k_bh_.

## 2. Materials and Methods

### 2.1. Source of Test Chemicals

All drugs were obtained locally, from the following suppliers: Rifampicin (Eremfat^®^ 300 mg, Riemser Pharma GmbH, Greifswald, Germany), ciclosporin (Sandimmun^®^ 50 mg/mL, Novartis Pharma GmbH, Nuremberg, Germany), ketoconazole (HRA 200 mg, HRA Pharma Deutschland GmbH, Wiesbaden, Germany), bosentan (Sigma-Aldrich, St. Louis, MO, USA), asunaprevir (asunaprevir, MedChemExpress, Monmouth Junction, NJ, USA), and pioglitazone (pioglitazone, Merck & Co., Inc., Rahway, NJ, USA repository).

### 2.2. Review of Inhibitory Potency and Model-Based Dose Selection for Drugs

In vitro inhibition constants (K_i_) and half maximum inhibitory concentrations (IC_50_) of the six drugs for human transporters OATP1B1, OATP1B3, NTCP, and MRP2 and their rat homologues (rOatp1a4, rOatp1b2, rNtcp, and rMrp2), were collated from literature sources and in-house measurements.

For all the selected drugs, compartmental pharmacokinetic models were developed to inform dose selection in the DCE-MRI studies. The one-compartment, two-compartment, or three-compartment pharmacokinetic models, following intravenous and oral administration, were fitted to published pharmacokinetic data for these drugs in rats. The models assumed linear pharmacokinetics of the six drugs investigated, to allow identification of the model parameters with the limited available rat pharmacokinetic data. Subsequently, simulations with the empirical compartmental models were undertaken, to identify doses that would result in free plasma concentrations in rats, during the timescale of the MRI, that were within the range of free steady-state plasma concentrations achieved following oral therapeutic drug dosing in humans. The model equations, sources of pharmacokinetic data, parameters estimates, and criteria for the dose selection for all the drugs are reported in the Supplementary Material, Section S1.

### 2.3. Animal Handling and In Vivo Study Design

In vivo studies were undertaken at three sites (D, E, G1 + G2) at two different field strengths, 4.7 T (D, G2) and 7 T (E, G1). Details of the equipment used are given in [25]. Animal procedures were compliant with directive 2010/63/EU or Institutional Animal Care and Use Committee (IACUC), for studies performed in the European Union or United States, respectively. Male Wistar rats, approximately 250 g body weight at the time of ordering, were locally obtained from Charles River Laboratories, allowed to acclimate for one week prior to study initiation, socially housed in 12 h light/dark cycles, and were provided standard rat chow and water ad libitum. All studies were performed between April 2018 and November 2019. Table 1 summarizes the drugs, dose, number of animals, and the time of dosing prior to the gadoxetate injection for each site. Details of vehicle formulation and preparation of drugs for intravenous injection are listed in Supplementary Material, Section S2, Table S8. No formal sample size calculation was performed: group sizes of 4–6 were chosen, consistent with previous work, balancing reduced animal use with the ability to detect substantial treatment effects. No blinding or randomization was performed.

Drug administration and MRI acquisition were performed on animals anesthetized by inhalation of isoflurane in an air mixture, approved by each institution’s animal committee, and maintained using approximately 2% isoflurane in the air mixture. Animals were monitored for respiratory rate and temperature, and a heating source, to maintain body temperature, was provided by each site. MRI was acquired in all animals on two separate days, which were separated by a washout interval of 48 h. On the first occasion, rats were dosed with vehicle (Table S8) via a tail vein catheter, using a drug-dose equivalent volume. After a drug-dependent interval (Table 1), rats were given gadoxetate (Primovist^®^ or Eovist^®^, Bayer AG, Berlin, Germany), diluted 1:5 in saline and administered at 0.5 mL/kg (25 μmol/kg) over 30 s via the tail vein. On the second occasion, the procedure was repeated with a drug.

### 2.4. Gadoxetate DCE-MRI Data Acquisition and Elaboration

When gadoxetate is co-administered with the vehicle, the contrast agent is rapidly taken up from blood plasma into liver parenchyma, then is effluxed via bile into the gastrointestinal tract. The DCE-MRI data enable quantitative analysis of time-dependent alterations in gadoxetate concentrations in the blood and liver [26]. The imaging setup and acquisition were identical to that reported previously [24]. The DCE-MRI sequence was acquired using a T_1_-weighted spoiled gradient echo sequence [17,27], with contrast agent administered after five baseline images had been acquired. Retrospective respiratory gating was employed during DCE-MRI data acquisition. On each occasion, 30 consecutive DCE-MRI measurements, including the five baseline images, were collected, at a 58 s temporal resolution, to capture the wash-in and wash-out of gadoxetate from the liver.

The derivation of DCE-MRI liver profiles employed the software PMI (Platform for research in Medical Imaging) v3.1 [28] at all the sites. DCE-MRI plasma profiles were also derived from data acquired from the spleen. Regions of interest (ROI), covering whole liver and spleen, were selected manually, as shown in Supplementary Material, Figure S10. Area under the curve (AUC_0-t_) for the ΔR_1_-time profiles for plasma and liver were calculated for each individual profile by using trapezoidal rule, integrating between the time of gadoxetate administration (t = 0) and the time of the last DCE-MRI measurement, where $R_{1}\equiv T_{1}^{-1}$.

### 2.5. PBPK Modelling and Prospective Hepatic Transporters DDI Prediction

**Figure 1:**
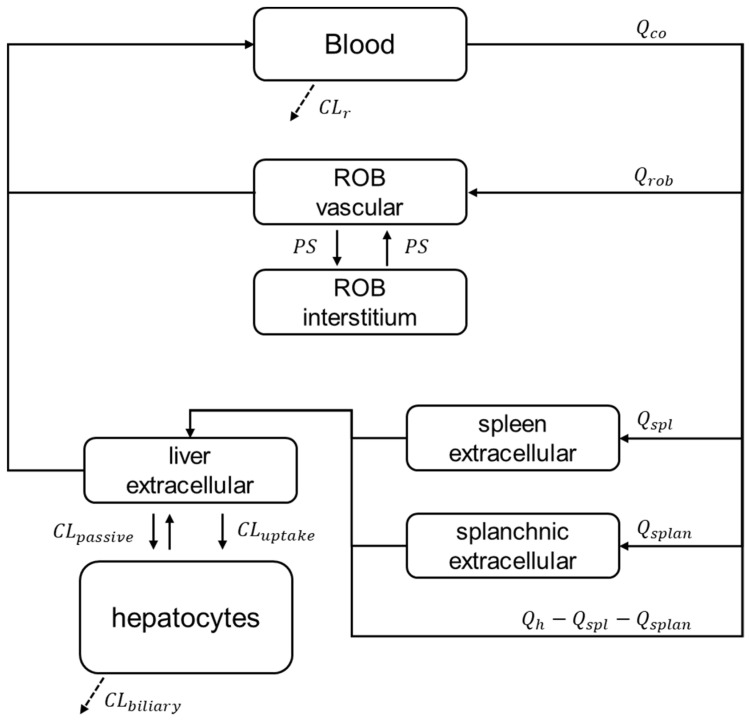
Structure of the reduced gadoxetate PBPK model. Continuous arrows represent the mass exchange within the system, while dashed arrows represent gadoxetate elimination. Subscripts co, rob, spl, splan, h, and r, represent cardiac output, rest of the body, spleen, splanchnic organs, hepatic and renal, respectively. CL, Q, and PS, represent the clearance processes, the blood flows, and the permeability surface product, respectively. Reproduced from [23] under CC-BY license.

A previously developed reduced PBPK model of gadoxetate [23] was used in the current study. The PBPK model (Figure 1) is composed of seven compartments: blood, spleen, splanchnic organs, liver interstitial space, hepatocytes, rest of the body (ROB) vascular, and extravascular space. In the ROB compartment, muscles, fat, bones, and skin, among others, are represented.

A permeability-limited liver model [29] was applied to describe the gadoxetate active uptake into the hepatocytes, as per Equation (1).

(1) $$\begin{array}{c} V_{liv,extr}\frac{dc_{liv,extr}}{dt}=input_{splan}+input_{art}-Q_{h}\frac{c_{liv,extr}}{K_{liv,\;extr-b}}-CL_{uptake}\cdot \;c_{liv,extr}-CL_{passive}(c_{liv,extr}-\\ f_{u,liv,\;cell}\;c_{liv,cell})\\ V_{liv,cell}\frac{dc_{liv,cell}}{dt}=CL_{uptake}\;c_{liv,extr}+CL_{passive}\left(c_{liv,extr}-f_{u,liv,\;cell}\;c_{liv,cell}\right)-CL_{biliary}\;f_{u,liv,cell}\;c_{liv,cell} \end{array}$$

$c_{liv,extr}$, $c_{liv,cell}$ [μmol/L] and $V_{liv,extr}$, $V_{liv,int}$ [L] are the gadoxetate concentrations (c) and volumes (V) of the liver extracellular space and hepatocytes, respectively. Considering the liver fenestrated capillaries, and that gadoxetate does not distribute into red blood cells, $V_{liv,extr}\;$was defined as the sum of liver plasma and interstitial volumes. $Q_{h}$ [L/h] is the sum of the portal vein and hepatic artery blood flow; $CL_{uptake}$, $CL_{passive}$, and $CL_{biliary}$ [L/h] are the active and passive uptake clearances across the hepatocyte sinusoidal cell membrane, and the excretion clearance from the hepatocytes to the bile, respectively; $f_{u,liv,cell}$ is the unbound fraction of gadoxetate in the hepatocytes; $K_{liv,\;extr-b}$ is the extracellular liver to blood partition coefficient; $input_{splan}$ and $input_{art}$ [μmol/h] are the portal vein and hepatic artery inputs to the liver, respectively. The model assumes no enterohepatic recirculation of gadoxetate following hepatobiliary excretion. Details of the model development, relations between concentrations in PBPK compartments and measured $\Delta R_{1}$, parameters identification, and performance were described previously [23].

The inhibition of $CL_{uptake}$ and $CL_{biliary}$ were prospectively predicted according to Equation (2) [30].

(2) $$CL_{transporter,inh}=\frac{CL_{transporter}}{1+c_{u,p}\left(t\right)/IC_{50}}$$

$CL_{transporter}$ and $CL_{transporter,inh}$ are the non-inhibited (baseline) and inhibited transporter intrinsic clearance (either $CL_{uptake}$ or $CL_{biliary}$); $c_{u,p}\left(t\right)$ is the plasma unbound concentration of the perpetrator at a given time t, simulated with the pharmacokinetic models (Supplementary Material, Section S1); $IC_{50}$ is the in vitro measure of the potency of the perpetrator in inhibiting the respective hepatic transporter (rOatp1b2 for active uptake, and rMrp2 for biliary efflux). IC_50_ and K_i_ data were collated from the literature and in-house measurements. When multiple sources were available, the prospective predictions used the lowest values of either IC_50_ or K_i_ to account for the worst-case scenario. Due to paucity of inhibition data for some of the drugs investigated for rat transporters, data obtained for the respective human transporters (OATP1B1 and MRP2) were used as a surrogate where necessary. The renal excretion clearance of gadoxetate ($CL_{r}$) was assumed to be unaffected by the test drugs. The performance of the prospective prediction using the PBPK modelling was evaluated by comparing the simulated gadoxetate AUC ratios (AUCR) in the plasma (derived from spleen compartment) and liver with the observed values. The AUCR is defined as shown in Equation (3).

(3) $$AUCR_{\tau }=\frac{AUC_{\tau ,\Delta R_{1},treated}}{AUC_{\tau ,\Delta R_{1},control}}$$

$AUC_{\tau ,\Delta R_{1},control}$ is the *AUC* of the gadoxetate $\Delta R_{1}$ in tissue τ when administered alone, while $AUC_{\tau ,\Delta R_{1},treated}$ is the equivalent when gadoxetate is administered following a given perpetrator. The AUCs were calculated from the administration of gadoxetate (t=0 min) until the end of the experiment (t=25 min).

When applied to $CL_{biliary}$, Equation (2) assumes that the unbound concentration of drug at the site of inhibition of hepatobiliary efflux transporter on the canalicular, is equal to the unbound concentration of the drug in plasma ($c_{u,p}$). Previous reports have shown that some of the drugs investigated here accumulate in hepatocytes (e.g., rifampicin, ciclosporin). Therefore, for selected drugs, an alternative inhibition model was explored, taking also into account their unbound concentration ratio between hepatocyte and plasma (Kpuu) (Supplementary Material, Section S4).

### 2.6. Tracer-Kinetic Model and K^trans^, k_he_, and k_bh_ Calculation

The tracer-kinetic model was developed to enable estimation of rate constants of hepatobiliary efflux from DCE-MRI profiles in individual animals. In this approach, the liver was assumed to consist of two compartments, the extracellular space (e) and the hepatocytes (h). Since water exchanges rapidly between those spaces, the change in relaxation rate was a weighted average (Equation (4)) [31,32].

(4) $$\Delta R_{1}\left(t\right)=r_{1,e}\cdot v_{e}\cdot c_{e}\left(t\right)+r_{1,h}\cdot v_{h}\cdot c_{h}\left(t\right)$$

The proportionality constant, $r_{1,\tau }$ $\left[L\cdot {\mathrm{mmol}}^{-1}\cdot s^{-1}\right]$, is the relaxivity of the contrast agent for the compartment τ (extracellular space, e or hepatocytes, h) at the respective field strength, while $v_{\tau }$ [mL/mL tissue] is the volume fraction of the compartment τ. Previously measured ex vivo $r_{1,tau}$ values for gadoxetate were used in this work [32], as per [23]. The underlying assumptions of the PBPK and tracer-kinetic models, relating to physiological volumes and perfusion, are consistent, despite different parameterizations according to the purpose of each model. For example, $V_{liv,extr}$ in the PBPK model is defined as an absolute volume (units = L; sum of liver plasma and interstitial volumes), while in the tracer-kinetic model, $v_{e}$ is a volume fraction (dimensionless), defined as $V_{liv,extr}/V_{liv\mathrm{er}}$, where $V_{liver}$ is the total liver volume. While some analogy can be drawn between $CL_{active}$ and k_he_, and $CL_{biliary}\;$and k_bh_, the interpretations are distinct. For example, the operating concentrations of the PBPK parameters $CL_{active}$ and k_he_, and $CL_{biliary}$ are the unbound concentrations of gadoxetate in plasma and hepatocytes, while the tracer-kinetic model is parameterized with respect to the total concentration of gadoxetate.

Since $c_{e}\left(t\right)$ is the input to the hepatocytes, and no backflux from hepatocytes to extracellular space is assumed, Equation (5) can be derived.

(5) $$v_{h}\cdot c_{h}\left(t\right)=e^{-\frac{t}{T_{h}}}*k_{he}\cdot c_{e}\left(t\right)$$

where *T_h_* is the mean transit time of gadoxetate in the hepatocytes [min], and * is convolution. The extracellular space is assumed to be in equilibrium with the blood pool and therefore the concentration *c_e_*(*t*) is proportional to the concentration *c_p_*(*t*) in the plasma of the feeding artery, i.e., *c_e_*(*t*) = (1 − *E*)·*c_p_*(*t*), where *E* is the extraction fraction of gadoxetate in the liver. Combining this assumption with Equations (4) and (5), and using (1 − *E*)·*k_he_* = *E·F_p_*, with *F_p_* being the apparent plasma flow into the liver [mL/min/mL tissue volume], gives Equation (6), the operational equation for gadoxetate DCE-MRI in the liver.

(6) $$\Delta R_{1}\left(t\right)=\left(1-E\right)\cdot v_{e}\cdot r_{1,p}\cdot c_{p}\left(t\right)+E\cdot F_{p}\cdot r_{1,h}\;\cdot e^{-t/T_{h}}\;*c_{p}\left(t\right)$$

Sensitivity analyses demonstrated that, with data sampled at 1 min time intervals, the amplitudes of both terms could not be measured separately, and therefore the fitted parameters were E and T_h_, whereas *v_e_* and *F_p_* were fixed to literature values. The rate constants were derived as *K^trans^* = *E·F_p_*, *k_he_* = *E·F_p_*/(1 − *E*), and *k_bh_* = *v_h_*/*T_h_*, with a literature value for *v_h_*. The plasma concentrations *c_p_*(*t*) were not reliably measured in small animals due to the small diameter of the main vessels compared to the resolution of the measurement. Therefore, spleen data were used in some instances, as a substitute (as per PBPK analysis), but these were found to be unreliable when applied for tracer-kinetic modelling in this study (not shown). Therefore, the source term *c_p_*(*t*) was derived from a two-compartment pharmacokinetic model for gadoxetate (see Supplementary Material, Section S5), and a step function as input in the blood compartment. The parameters k_bh_ and k_he_ were fitted to the liver data using a model implementation in Python [33]. For each rat considered in these studies, the rate constants K^trans^, k_he_, and k_bh_ were calculated, in both control and inhibitory phases. The rate constants of the control and inhibitory phases were compared via calculation of simple (unstandardized) effect sizes (i.e., simple effect size = μ_control_ − μ_treatment_, where μ is the mean average of all subjects) at the 95% confidence level. Statistical significance was assessed through a two-sided paired t-test, with *p* < 0.05 considered significant.

## 3. Results

**Figure 2:**
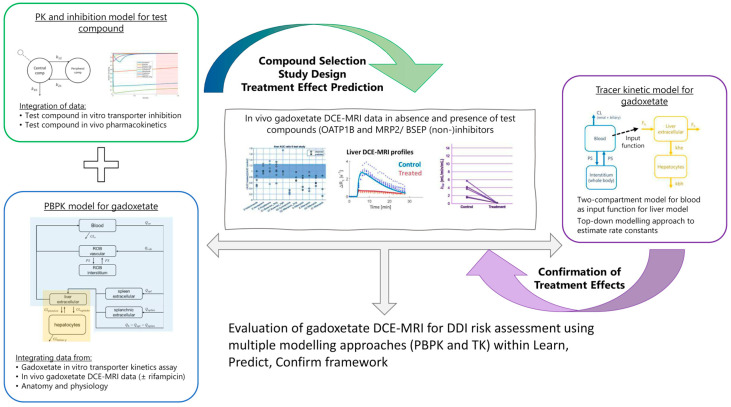
Framework for gadoxetate dynamic contrast enhanced magnetic resonance imaging (DCE-MRI)-based evaluation of hepatobiliary drug–drug interactions (DDI). The framework embeds modelling and simulation techniques, including physiologically-based pharmacokinetic (PBPK) and tracer-kinetic (TK) modelling throughout the study, from study design through to data analysis.

In the current study, an integrated framework of in silico study design and model-based analysis was applied for gadoxetate DCE-MRI evaluation of hepatobiliary DDI in rats (Figure 2).

### 3.1. Drug Inhibitory Potency and Model-Based Dose Selection

In vitro inhibition data (e.g., IC_50_ and K_i_) from the literature, for rat hepatobiliary transporters rOatp1a4, rOatp1b2, rNtcp, and rMrp2 are summarized in Table 2, while the corresponding data for human OATP1B1, OATP1B3, NTCP, and MRP2 transporters are summarized in Table 3. In vitro inhibition data in rats were available for rifampicin and ciclosporin, but were scarce for the other drugs, whereas data were available in most cases for the human transporters. For the limited number of transporters where in vitro inhibition potencies were reported in both rat and human, data were generally consistent, with the exception of the ciclosporin IC_50_ for rOa1p1a4 and rOatp1b2, which were higher than reported values for this drug with the human OATP1B transporters.

The published OATP1B1 and/or OATP1B3 IC_50_ values of bosentan, pioglitazone, asunaprevir, and ketoconazole were lower than the unbound plasma concentrations achieved at steady state following oral therapeutic administration of the drugs in humans. As there were no data for rOatp1 transporters for these drugs, no OATP1B DDI was anticipated. Conversely, based on in vitro data, rifampicin and ciclosporin were expected to inhibit rOatp1/OATP1B, supported also by clinical evidence of OATP1B mediated DDIs [30,34]. Two drugs (ciclosporin and pioglitazone) also exhibited similar potencies of NTCP inhibition (Table 3).

**Table 2:** In vitro inhibition constants (K_i_ [µM]) and half maximal inhibitory concentrations (IC_50_ [µM]) for selected drugs, against a range of rat hepatic uptake and biliary efflux transporters. Data were extracted from published literature [35,36,37,38,39,40,41,42].

Drug	K_i_ (µM) rOatp1a4	IC_50_ (µM) rOatp1a4	IC_50_ (µM) rOatp1b2	IC_50_ (µM) rOatp1b2	IC_50_ (µM) rNtcp	IC50 (µM) rMrp2
Rifampicin	2.9	1.3	0.79	0.6–1.1	NA	20–53
Asunaprevir	NA	NA	NA	NA	0.6	11
Bosentan	NA	NA	NA	NA	0.4	NI
Ciclosporin	NA	3–30	1.2	NA	1.5	5–15
Ketoconazole	NA	NA	NA	NA	NA	NA
Pioglitazone	NA	NA	NA	NA	NA	NA

NA—no data available; NI—reported not to inhibit the transporter.

**Table 3:** In vitro inhibition constants (K_i_) and half maximal inhibitory concentrations (IC_50_) for selected drugs, against a range of human hepatic uptake and biliary efflux transporters and respective maximum unbound drug plasma concentrations (C_max,u_) in humans. Data were extracted from published literature [30,43,44,45,46,47,48,49], regulatory review and prescribing documents [50,51,52,53,54,55], and in-house data from Merck & Co., Inc., Rahway, NJ, USA.

Drug	K_i_ (µM) OATP1B1 ^a^	IC_50_ (µM) OATP1B1 ^a^	IC_50_ (µM) OATP1B3	IC_50_ (µM) NTCP	K_i_ (µM) MRP2 ^a^	IC_50_ (µM) MRP2 ^a^	C_max_ (µM) [Daily Dose]	fu
Rifampicin	0.67 (0.22–17)	1.90 (0.24–120)	0.11	277	24.3 (7.9–40.6)	55 (14.7–144)	0.85 [600 mg]	0.2
Asunaprevir	NA	0.55 (0.3–0.79)	0.65	NA	NA	4	0.76 [200 mg]	0.012
Bosentan	NA	6.6 (5.0–8.2)	5.2	18	NA	>100	3.3 [250 mg]	<0.02
Ciclosporin	0.014 (0.22–2.32)	0.50 (0.02–3.5)	0.032	0.37	4.7 (21–24)	2.7 (5.6–45.3)	1.5 [4 mg/kg]	0.1
Ketoconazole	50.7 (11.5–107.7)	15.4 (1.8–60.9)	3.9	202	NA	>20	6.6 [200 mg]	0.01
Pioglitazone	NA	5.09 (11.1–39.6)	3.41	4.04	NA	>133	4.8 [30 mg]	<0.01

^a^ Key transporters for gadoxetate hepatobiliary disposition.

### 3.2. DCE-MRI Interaction Data

The majority of rats used in the procedures survived until the end of the study without adverse effects. Higher doses of bosentan (4–6 mg/kg) were explored, but were not tolerated well and therefore the study was discontinued, in accordance with ethics, due to adverse effects. In this study, n = 3 animals received 4 mg/kg bosentan, and n = 1 received 6 mg/kg, and contributed data to the final analysis. An ROI could not be obtained for the spleen in the animal that received 6 mg/kg. Data from two additional animals were not analyzable (n = 1 from asunaprevir study; n = 1 from ketoconazole study) and therefore did not contribute to the final analyses.

**Figure 3:**
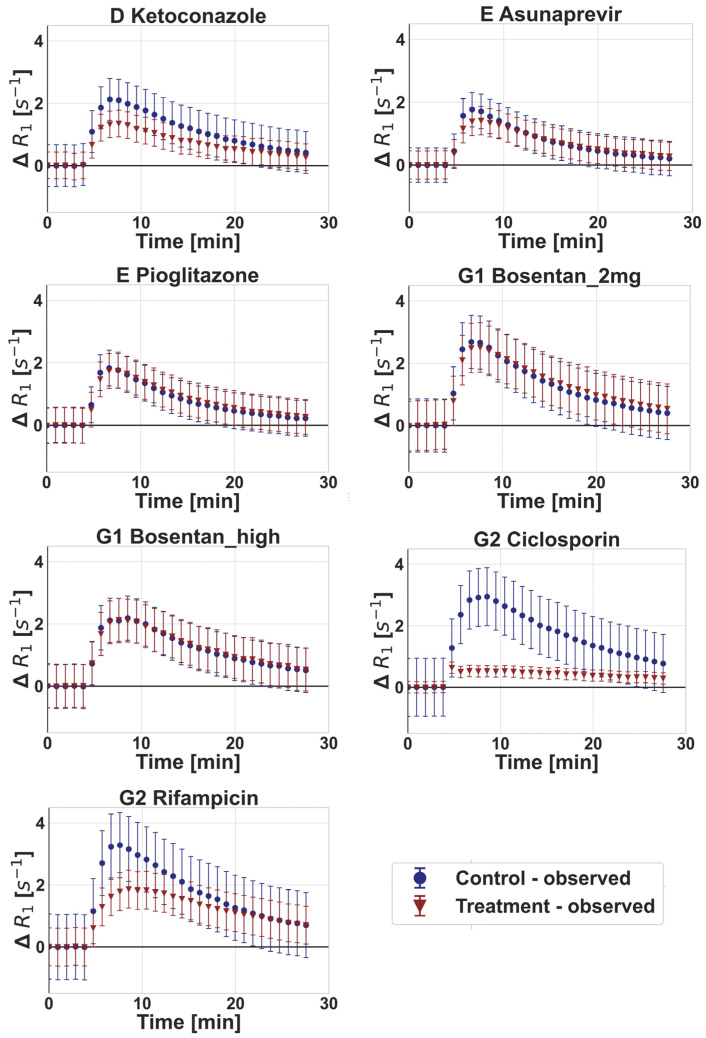
Observed gadoxetate liver profiles in control phase and following administration of drugs (treatment) at different sites (D, E, G1, and G2). Symbols and error bars represent mean and standard deviation (between 3 and 6 animals), respectively. Doses for each drug are listed in Table 1, where Bosentan_2mg and Bosentan_high refer to the 2 mg/kg and 4–6 mg/kg doses of bosentan, respectively.

**Figure 4:**
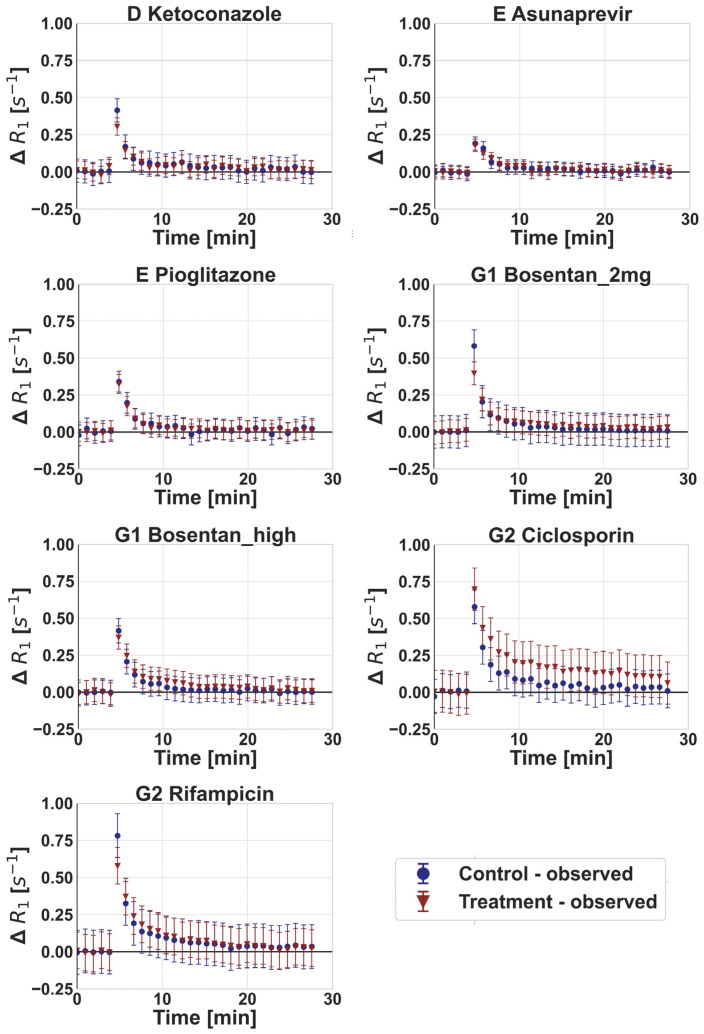
Observed gadoxetate plasma profiles in control phase and following administration of drugs (treatment) at different sites (D, E, G1, and G2). Symbols and error bars represent mean and standard deviation (between 3 and 6 animals), respectively. Doses for each drug are listed in Table 1, where Bosentan_high refers to the 4–6 mg/kg doses of bosentan.

Ciclosporin and, to a lower extent, rifampicin (2 mg/kg) and ketoconazole were associated with a decrease in maximum ΔR_1_ and AUC of gadoxetate ΔR_1_ in the liver compared with the vehicle control, while no relevant changes were noted for any of the other drugs (Figure 3). The plasma and liver gadoxetate AUCR for all the drugs investigated in this study are reported in Table 4, including also data following a rifampicin dose of 10 mg/kg, as reported previously [24]. Reduced active uptake of gadoxetate into the liver in the presence of some of the inhibitors resulted in a corresponding increase in gadoxetate exposure in the plasma (Figure 4). For example, ciclosporin caused a 1.94-fold increase in gadoxetate plasma, AUC (1.57–3.38) and median fold decrease in gadoxetate liver AUC of 3.85 (3.7–5). Similar results were obtained for rifampicin dosed at 10 mg/kg. In contrast, rifampicin dosed at 2 mg/kg showed a weaker effect on both liver and plasma AUCs. All the other drugs showed a marginal effect on gadoxetate plasma and liver AUCs, with the exception of ketoconazole and a high dose of bosentan (4–6 mg/kg). Interestingly, the study arm treated with ketoconazole showed a median AUCR for plasma and liver equal to 0.68 (0.38–0.87) and 0.52 (0.47–0.84), respectively. Across all drugs and studies, the AUCR had a moderate to high inter-individual variability, with a median (range) coefficient of variation (%CV) of 33% (14–103%) and 26% (8–64%) for plasma and liver, respectively (Table 4). These results, with the exception of ketoconazole, are in agreement with the inhibitory potency reported in the literature for these drugs, and the simulations of the inhibited fraction by individual transporters performed with the empirical compartmental model for the drugs (see Supplementary Material, Section S1).

### 3.3. Prospective Prediction of Gadoxetate Hepatic Transporter-Mediated DDIs with PBPK Model

**Figure 5:**
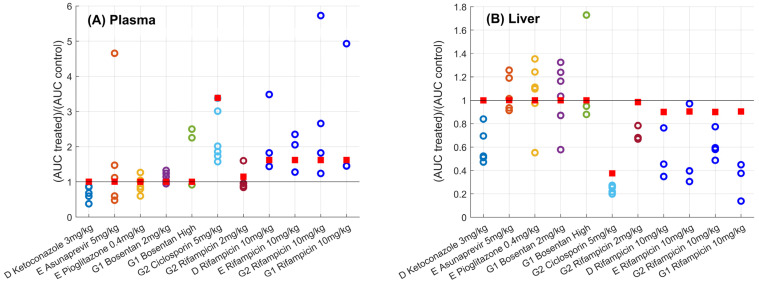
Predicted (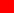) versus observed (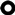) plasma (Panel **A**) and liver (Panel **B**) AUCR for different sites (D, E, G1, and G2) and drugs. Solid black line corresponds to the line of unity. Predictions using the gadoxetate physiologically-based pharmacokinetic model considered inhibition of both $CL_{active}$ and $CL_{biliary}$, assuming unbound plasma concentrations to drive inhibition of each transporter of interest. AUCR = ratio of treated vs. control area under the curve of gadoxetate $\Delta R_{1}$-time profiles (Equation (3)). Doses for each drug are listed in Table 1; “Bosentan_high” refers to the 4–6 mg/kg doses of bosentan.

**Figure 6:**
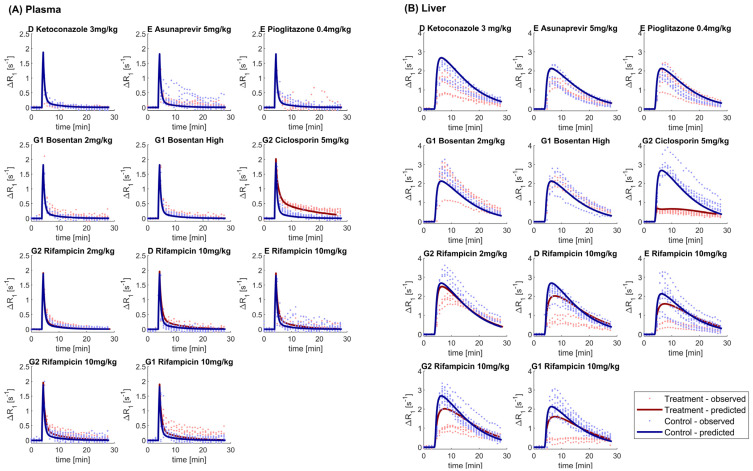
Predicted versus observed ΔR_1_-time profiles for different sites (D, E, G1, and G2) and perpetrators (asunaprevir, bosentan, ciclosporin, ketoconazole, pioglitazone, and rifampicin) in plasma (Panel **A**) and liver (Panel **B**). Predictions using the gadoxetate physiologically-based pharmacokinetic model considered inhibition of both $CL_{uptake}$ and $CL_{biliary}$, assuming unbound plasma concentrations to drive inhibition of each transporter parameter. Doses for each drug are listed in Table 1; “Bosentan High” refers to the 4–6 mg/kg doses of bosentan.

Gadoxetate liver and plasma AUCR were prospectively predicted using the PBPK model for this imaging biomarker, coupled with the pharmacokinetic models for all the considered perpetrators. Comparison of predicted versus observed gadoxetate AUCR for the plasma and liver are reported in Table 4 and in Figure 5, respectively. Predicted versus observed $\Delta R_{1}$ profiles in plasma and liver are shown in Figure 6.

The PBPK model predicted well the gadoxetate $\Delta R_{1}$ plasma profiles in the control phase (Figure 6A). The between-site variability was limited and therefore the mean profile simulated with the PBPK model was sufficient to capture the systemic gadoxetate pharmacokinetics. When gadoxetate was co-administered with ciclosporin and rifampicin, the inhibition of gadoxetate liver uptake clearance was accompanied by an increase in $\Delta R_{1}$ in the terminal phase of the plasma profile, which was predicted successfully by the PBPK model (Figure 6A). The PBPK model coupled with the perpetrators compartmental models, predicted reasonably well the change in gadoxetate plasma AUC for ciclosporin, and lack of change in AUC for asunaprevir, pioglitazone, and bosentan. This approach also captured the effect of rifampicin as a function of the dose: for a 2 mg/kg dose the model correctly predicted a lack of interaction (AUCR<1.25) and increase in the DDI at 10 mg/kg, although the magnitude was under-predicted (predicted AUCR=1.62 vs. 1.89; average of four studies, Table 4).

Gadoxetate liver profiles in the control phase showed a high between-site variability, in agreement with previous reports [24]. As a result, PBPK modelling either slightly underpredicted or overpredicted the control liver profiles (Figure 6B), depending on the observed data used for comparison. PBPK modelling was able to distinguish negative controls from the OATP1B inhibitors, e.g., it predicted correctly a strong inhibition and change in gadoxetate liver AUC in the presence of ciclosporin and no inhibition in the case of asunaprevir, pioglitazone, and bosentan. Conversely, for ketoconazole, the PBPK model predicted no effect on gadoxetate liver AUC, in contrast to the observed decrease in the liver AUC (Figure 5). For rifampicin, the PBPK model tended to underestimate the extent of the effect on gadoxetate liver AUC, as the predicted liver AUCR was between 1.4- and 2.4-fold higher than the median of those observed. A similar tendency, but to a lower extent, was noted for ciclosporin, where a 1.5-fold difference was seen between the predicted and observed AUCR.

### 3.4. Tracer-Kinetic Model Based Analysis

**Figure 7:**
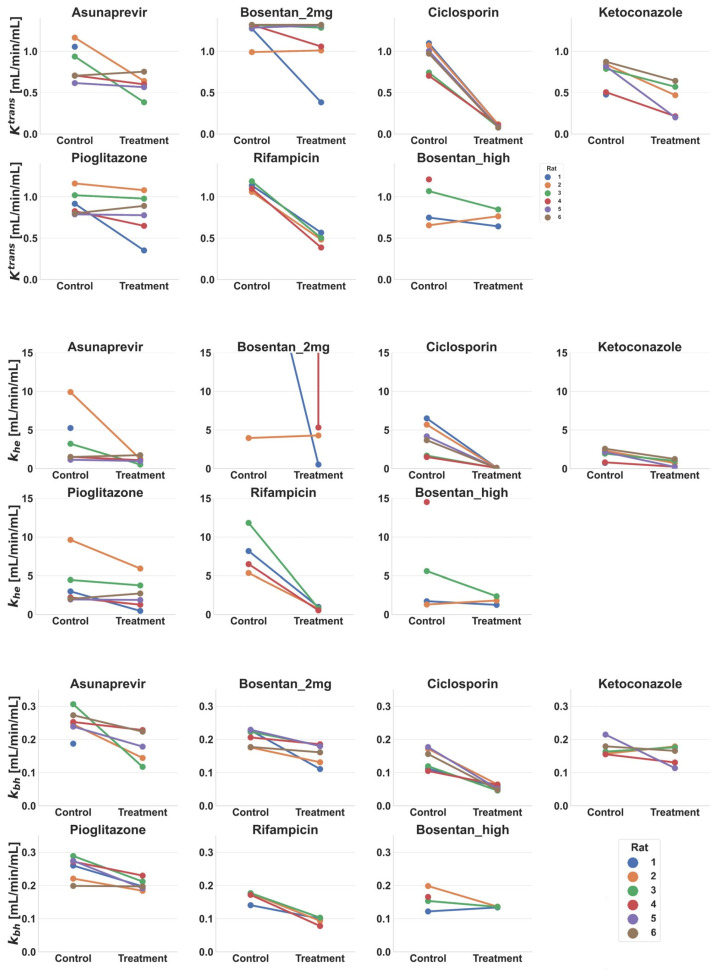
Effect of drug administration on estimated tracer-kinetic model rate constants for hepatic plasma clearance (K^trans^), hepatic gadoxetate uptake (k_he_), and biliary excretion (k_bh_), for different sites (D, E, G1, and G2) and drugs. Each line represents a single rat. Doses for each drug are listed in Table 1, where Bosentan_high refers to the 4–6 mg/kg doses of bosentan.

The effects of the six drugs on K^trans^, k_he_, and k_bh_ of gadoxetate in rats in vivo, as estimated via the tracer-kinetic modelling, are shown in Figure 7. Simple effect sizes calculated from these values are shown in Table 5.

Following administration of ciclosporin, a marked and statistically significant de-crease was observed on average in gadoxetate K^trans^ (−89%, *p* < 0.001), k_he_ (−96%, *p* = 0.006), and k_bh_ (−59%, *p* = 0.002). To a lesser extent, statistically significant decreases in gadoxetate parameters were observed following administration of rifampicin, with changes in K^trans^ (−57%, *p* < 0.001), k_he_ (−90%, *p* = 0.01), and k_bh_ (−43%, *p* = 0.008). In the case of ketoconazole, decreases in K^trans^ (−46%, *p* = 0.009) and k_he_ (−65%, *p* = 0.006) were apparent, whereas no significant changes were detected in k_bh_ (*p* = 0.39). Statistically significant reductions were also observed in k_bh_ following dosing to rats of pioglitazone (*p* = 0.01) and asunaprevir (*p* = 0.04), however this was not observed in K^trans^ or k_he_ for these drugs.

No statistically significant effects on either K^trans^ or k_bh_ were detected following administration of either of the bosentan doses. This trend was also evident for k_he_ following administration of the bosentan high dose. A firm conclusion could not be obtained regarding changes in k_he_ following administration of the bosentan 2 mg/kg dose, as physiologically plausible values over both control and treatment days were only obtained for one animal (k_he_ > 37 mL/min/mL for all other animals).

## 4. Discussion

A major challenge for prediction of transporter-mediated clinical DDIs is poor translation between in vitro transporter inhibition data and in vivo functional effects. In such instances, plasma DDI data, or interaction data with endogenous biomarkers for transporters of interest, have been used to refine PBPK model parameters [34,56]. However, verification of the predicted changes in tissue exposure as a result of transporter modulation remains a challenge. The current study effectively incorporated modelling and simulation techniques into the design and data analysis of gadoxetate DCE-MRI experiments to enable quantification of the effects of multiple tests-drugs on the function of hepatic OATP1B and MRP2 transporters in vivo (Figure 2).

### 4.1. Data Analysis and Endpoints for Transporter Interaction Assessment with Imaging Data

The application of imaging modalities for evaluation of tissue exposure and local pharmacokinetic measurement (e.g., in liver, brain, and tumors), has shown promising results [23,57,58,59,60,61,62]. In the current study, an assessment of drug effects on gadoxetate was performed, using both non-compartmental analysis (i.e., AUCR of $\Delta R_{1}$ profiles in liver and plasma), and tracer-kinetic modelling. Alternatively, integration plot analysis may be applied for analysis of kinetic imaging data, but this approach has a number of limitations, including (i) the need for imaging data from the bile duct or intestine (not measured in current study), (ii) the very rapid uptake phase of gadoxetate in the liver and elimination from plasma (Figure 3 and Figure 4), and (iii) an inability to differentiate roles of blood flow and transporter activity with respect to hepatic uptake. The tracer-kinetic and PBPK models applied in the current study overcame these limitations.

**Figure 8:**
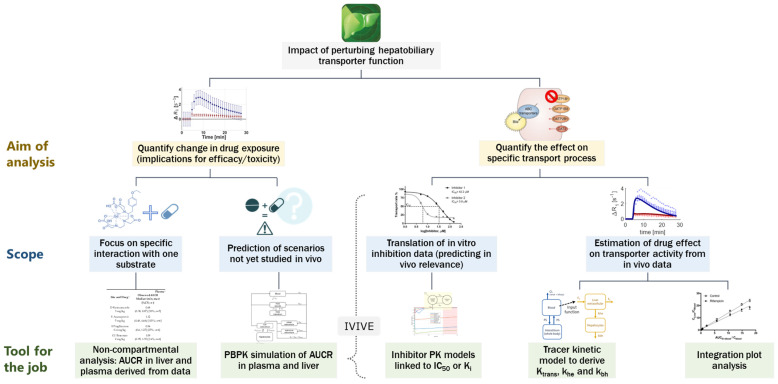
Scheme showing the applications for the data analysis ‘toolkit’ for quantitative evaluation of perturbations in transporter kinetics from imaging data.

Assessment of changes in the victim drug AUC (AUCR), is a standard endpoint for evaluation of the magnitude of DDI in drug development, representing the net effect of the interaction on the exposure in the plasma [63,64] (Figure 8). AUCR (either clinically observed or predicted by PBPK modelling) is often used to inform dosage adjustment (or contraindication) recommendations for clinical practice, involving the specific pair of co-administered drugs. In the current study, the PBPK predictions of AUCR, based on IVIVE of in vitro transporter inhibition data, tended to under-predict the magnitude of gadoxetate interactions when compared with the in vivo data in the liver, but showed reasonable predictive performance for plasma data (Figure 5). A limitation of net-effect parameters, such as plasma or liver AUCR, is that the perturbations of specific processes (e.g., uptake and efflux) may not be readily delineated from the analysis of the observed data. In contrast, PBPK and tracer-kinetic modelling of the DCE-MRI data can estimate drugs’ effects on both transporters mediated uptake (i.e., k_he_ or $CL_{active}$) and efflux (k_bh_ or $CL_{biliary}$). As an example, the median decrease in gadoxetate liver AUC, by 74%, was noted in the presence of ciclosporin compared to the control (Table 4). The uptake rate (k_he_) was estimated to decrease by 96% using the tracer-kinetic model (Table 5), while IVIVE-PBPK simulations predicted between 97% and 98% inhibition of $CL_{active}$ during the gadoxetate DCE-MRI period. Therefore, for the purpose of quantifying the perturbation effects of inhibitor drugs on specific transport processes, model-based analysis of imaging data is recommended.

### 4.2. Integrative Approach Needed to Interpret Imaging Data

In this work, the PBPK model, coupled with the drugs’ pharmacokinetic models, predicted the changes in gadoxetate plasma AUC, following coadministration of the six drugs investigated in this study, with reasonable accuracy. In the case of liver, the model could distinguish the negative controls from the OATP1B inhibitors. The exception was ketoconazole which caused a decrease in gadoxetate AUC compared with control both in plasma (1.5-fold) and in the liver (2-fold). The in vitro OATP1B1 IC_50_ (>2 µM; Table 3) for ketoconazole is relatively high compared with its plasma exposure (unbound C_max_ ~0.07 µM), and hence in vivo inhibition of hepatic uptake transport was not expected for this drug. rNtcp/NTCP was also reported to transport gadoxetate; inhibition of rNtcp by ketoconazole is also unlikely, considering the high IC_50_ for NTCP (202 µM, Table 3). Plausible explanations of the unexpected gadoxetate AUC observations with ketoconazole include instrument or signal-to-$\Delta R_{1}$ errors, an altered volume of interstitial space in the spleen (which was used to derive the plasma DCE-MRI profile) between the two phases of the study, or dosing error in either of the two occasions. An acute dose of ketoconazole is not expected to affect renal function parameters, such as glomerular filtration rate. Although some inhibitory interaction with rOatp4c1 in the kidney (expressed on the apical membrane of rat kidney [65]) could be speculated, there is no evidence to support it. The tracer-kinetic model estimated a 46% decrease in gadoxetate K^trans^ for ketoconazole, and when applied to the extended clearance concept paradigm [66], this decrease in K^trans^ should cause an increase in plasma AUC, the opposite to the observed data. This apparent paradox is likely explained by the use of a population mean two-compartment pharmacokinetic model for gadoxetate as input function for the tracer-kinetic model. As such, in the case of ketoconazole, it is unlikely that the estimated decrease in K^trans^ and k_he_ is reflecting a true inhibition of rOatp1b transporter activity, highlighting the importance of a holistic approach to interpretation of imaging data, considering the interplay of biological and technical systems.

### 4.3. Biological Relevance of Interaction Effects on K^trans^, k_he_, and k_bh_

Quantifying the effects on uptake and efflux transporters could provide valuable insights into DDI affecting drugs whose pharmacology targets are expressed within hepatocytes, e.g., statins. Insight can also be attained on the effects on bilirubin clearance that are not due to overt liver injury, or inhibition of glucuronidation. When normalized to the same units and reference concentrations, transport rate parameters in the PBPK and tracer-kinetic models were within 4-fold of one another, indicating reasonable consistency at the quantitative level (Table S11). Rifampicin and ciclosporin markedly inhibited gadoxetate uptake activity in vivo, based upon k_he_ in the control and inhibitory phases (Figure 7 and Table 5), in agreement with clinical OATP1B-mediated DDIs [6]. Ketoconazole has well-reported hepatotoxicity [67], but was not expected to result in OATP1B-mediated interaction in vivo [68] (Table 3).

Differentiation of K^trans^ and k_he_ has been overlooked in previous applications of tracer-kinetic modelling of gadoxetate DCE-MRI data in rats [17,27], but was important to consider in the current study, where the aim was to quantify treatment effects on rate constants, and (i) the establish biological relevance of the changes, and (ii) compare these across drugs. In particular, the hepatic extraction ratio (E) for gadoxetate in rats can be high in the absence of drugs (e.g., E > 0.95); in these cases, derived K^trans^ values approach the fixed liver perfusion rate and the system becomes perfusion-limited, making quantitative estimation of drugs’ effects on hepatic uptake (k_he_) highly conditional on the assumed value for liver perfusion. This scenario was evident for the bosentan 2 mg/kg dose, where physiologically implausible values were observed for k_he_, highlighting the necessity to consider both K^trans^ and k_he_ in data interpretation. The high extraction scenario limits the ability to detect hepatic rOatp1b inhibition in rats for weaker inhibitors. In humans, gadoxetate hepatic extraction is lower than in rats (approx. 20% [20,69,70]), and therefore would be of less concern for gadoxetate-based detection of OATP1B/MRP2-mediated DDIs. It is therefore recommended to consider both K^trans^ and k_he_ when considering the biological relevance of treatment effects on rate constants in rats.

Quantifying the effects on rMrp2 could be especially valuable, since this could result in concentrations of drugs and metabolites within the liver which differ with the ones in plasma [6,59]. For all six drugs investigated here, the largest effects were observed on the inhibition of active uptake. In addition to uptake parameters (K^trans^ and k_he_), gadoxetate DCE-MRI data, rifampicin and ciclosporin reduced k_bh_, suggesting inhibition of biliary efflux, while asunapravir and pioglitazone were associated with only reduced k_bh_. As expected, reduced K^trans^ and k_he_ were associated with decreased gadoxetate liver AUC in the presence of rifampicin and ciclosporin. In contrast, while reduced k_bh_ would be expected to be associated with an increase in liver AUC [59], this was not the case for asunaprevir (31% decrease in k_bh_, liver AUCR = 1.01) or pioglitazone (19% decrease in k_bh_, liver AUCR = 1.1).

### 4.4. Imaging Data to Support PBPK-Based Quantitative Translation

The PBPK modelling predicted well gadoxetate liver and plasma AUCR in the presence of ciclosporin, but under-predicted the liver AUCR in the case of rifampicin, despite a reasonable prediction in plasma (Figure 5). Possible contributing reasons for this mismatch between the plasma and liver predictive performances for rifampicin include, model assumptions such as the relaxivities and the assumption of fast water exchange, a pragmatic approach to translation of in vitro transporter IC_50_ and K_i_ data for efflux transporters, and the differential sensitivity of each tissue to uptake transporter inhibition.

In vitro measurements of IC_50_ and K_i_ are highly variable (e.g., Table 3), and are typically higher than corresponding model-based in vivo estimates [34,71]. Such trends are consistent with underestimation of the magnitude of inhibition of $CL_{active}$ and liver AUCR noted for rifampicin (Figure 6B). Likewise, there are limited examples of translation of in vitro inhibition data for hepatobiliary efflux transporters (e.g., MRP2). The assumption that the inhibitory concentration affecting gadoxetate $CL_{biliary}$ was equal to the unbound drug concentration in plasma, predicted < 25% inhibition of $CL_{biliary}$ using the median ciclosporin in vitro IC_50_ for MRP2 (Figure S11). Although commonly applied, such assumptions for the operating inhibitory concentrations for hepatobiliary efflux transporters have limitations, as these transporters do not face the plasma, highlighting a need for more mechanistic inhibitor models, which was beyond the scope of the current work. Nevertheless, corresponding PBPK predictions of gadoxetate in the presence of ciclosporin were not inconsistent with the observed data, and previous studies suggest that in vivo inhibition of rMrp2/MRP2 by ciclosporin may be modest [30,72]. An attempt to simulate the inhibition of $CL_{biliary}$ using the estimated unbound concentration in the hepatocyte (considering inhibitor liver Kpuu) overestimated the degree of this interaction (75–82% inhibition), based upon the gadoxetate DCE-MRI profile and the analysis using the tracer-kinetic model (59% decrease in k_bh_) (Figure 7 and Table 5).

In the case of gadoxetate, plasma and liver are expected to have differential sensitivity to rOatp1b2 inhibition. Whereas transporter-mediated uptake is the predominant contributing mechanism to gadoxetate uptake into hepatocytes [23], elimination of gadoxetate from systemic circulation is by both hepatic and renal elimination. As such, the liver AUC will be more sensitive to transporter inhibition compared with plasma. It is easier for a PBPK model prediction to appear successful when AUCR is close to 1 (e.g., lack of DDI, as in the case of plasma for 2 mg/kg rifampicin) [73]. Therefore, availability of liver imaging data provided further insight into the under-prediction of inhibition of hepatic uptake transport, which could not be obtained by an assessment of the DDI data in plasma alone. This finding reinforces the advantage of the liver imaging data for evaluating predictions of transporter DDIs. Upon translation to the clinical setting, imaging biomarker data can hence facilitate future refinement of PBPK models of hepatobiliary transporter inhibitors, to enhance the current paradigm of using a PBPK-projection of DDI magnitude in untested scenarios to support clinical study design and labelling recommendations [10,63].

## 5. Conclusions

In the current work, gadoxetate DCE-MRI imaging biomarkers (K^trans^, k_he_, and k_bh_) were demonstrated as promising for the detection and quantification of DDI via rOatp1b/rMrp2, in a preclinical setting, to identify compounds for which clinical DDI studies could be prioritized or deprioritized. Our preclinical studies show the potential to integrate PBPK modelling and tracer-kinetic models of gadoxetate DCE-MRI data in the framework of drug development (Figure 8).

Gadoxetate PBPK models (especially if extended to humans) are useful for translating in vitro transporters’ inhibition data of investigational new drugs, and prospective prediction of DDI risks and modulation of liver exposure, in support of other existing modelling tools/biomarkers. In future work, we will test and extend this integrative approach to humans, by analyzing clinical gadoxetate DCE-MRI profiles with both PBPK and tracer-kinetic modelling approaches.

## Figures and Tables

**Table 1:** Summary of gadoxetate imaging studies with six selected drugs in rats.

Drug	Dose	Number of Animals	Dose Staggering Time ^a^ (min)	Site (Field Strength) ^b^
Rifampicin	2 mg/kg	4	60	G2 (4.7 T)
Asunaprevir	5 mg/kg	6	30	E (7 T)
Bosentan	2 mg/kg	6	60	G1 (7 T)
Bosentan	4–6 mg/kg ^c^	4 ^c^	60	G1 (7 T)
Ciclosporin	5 mg/kg	6	60	G2 (4.7 T)
Ketoconazole	3 mg/kg	6	30	D (4.7 T)
Pioglitazone	0.4 mg/kg	6	30	E (7 T)

^a^ Time delay between dose of drug and administration of gadoxetate, informed by pharmacokinetic analysis and modelling of plasma concentration-time profiles of drugs investigated (see Supplementary Material, Section S1). ^b^ Sites are aligned with [25]; ^c^ higher dose of bosentan was explored but not tolerated well, therefore the study was discontinued, in accordance with ethics, due to adverse effects.

**Table 4:** Observed and physiologically-based pharmacokinetic model (PBPK) based prediction of the ratio of gadoxetate area under the curve, based on $\Delta R_{1}$, in the presence of investigated drugs relative to the vehicle control (AUCR), in plasma and liver.

Site and Drug ^a^	Plasma ^b^		Liver	
	Observed AUCR Median (min, max) [%CV; n ^c^]	Predicted AUCR	Observed AUCR Median (min, max) [%CV; n ^c^]	Predicted AUCR
D Ketoconazole 3 mg/kg	0.68 (0.38, 0.87) [30%; n = 5]	1.00	0.52 (0.47, 0.84) [26%; n = 5]	1.00
E Asunaprevir 5 mg/kg	1.12 (0.48, 4.66) [103%; n = 6]	1.01	1.01 (0.91, 1.26) [15%; n = 6]	1.00
E Pioglitazone 0.4 mg/kg	0.94 (0.6, 1.27) [25%; n = 6]	1.00	1.1 (0.55, 1.35) [26%; n = 6]	1.00
G1 Bosentan 2 mg/kg	1.09 (0.95, 1.33) [14%; n = 6]	1.00	1.1 (0.58, 1.32) [27%; n = 6]	1.00
G2 Bosentan 4–6 mg/kg	2.25 (0.92, 2.5) [45%; n = 4]	1.00	0.95 (0.88, 1.73) [40%; n = 4]	1.00
G2 Ciclosporin 5 mg/kg	1.94 (1.57, 3.38) [33%; n = 6]	3.39	0.26 (0.2, 0.27) [12%; n = 6]	0.38
G2 Rifampicin 2 mg/kg	0.92 (0.84, 1.6) [33%; n = 4]	1.15	0.68 (0.67, 0.78) [8%; n = 4]	0.98
D Rifampicin 10 mg/kg [24]	1.82 (1.44, 3.48) [48%; n = 3]	1.62	0.45 (0.35, 0.76) [41%; n = 3]	0.90
E Rifampicin 10 mg/kg [24]	2.06 (1.28, 2.35) [29%; n = 3]	1.62	0.4 (0.31, 0.97) [64%; n = 3]	0.90
G2 Rifampicin 10 mg/kg [24]	2.24 (1.24, 5.73) [70%; n = 4]	1.62	0.59 (0.49, 0.77) [20%; n = 4]	0.90
G1 Rifampicin 10 mg/kg [24]	1.45 (1.45, 4.93) [77%; n = 3]	1.62	0.38 (0.14, 0.45) [50%; n = 3]	0.90

^a^ Letters indicate the site of the study, as detailed in [25]; ^b^ derived from DCE-MRI data acquired in spleen, see methods. ^c^ Coefficient of variation (%CV) [standard deviation/mean] and number (n) of animals.

**Table 5:** Absolute effects of drugs on hepatic plasma clearance (K^trans^), hepatic uptake (k_he_), and biliary efflux (k_bh_) of gadoxetate as estimated using the tracer-kinetic model. Any statistically meaningful changes (where zero falls outside of the 95% CI) are marked using bold font.

	Simple Effect Size (95% CI) [mL/min/mL liver] ^a^		
Site and Drug ^b^	K^trans^	k_he_	k_bh_
D Ketoconazole 3 mg/kg ^c^	0.35 ** (0.20, 0.49)	1.27 ** (0.81, 1.74)	0.02 (−0.02, 0.06)
E Asunaprevir 5 mg/kg ^c^	0.24 (−0.01, 0.48)	2.34 (−0.92, 5.60)	0.09 * (0.03, 0.14)
E Pioglitazone 0.4 mg/kg	0.13 (−0.05, 0.32)	1.21 (−0.10, 2.51)	0.05 ** (0.03, 0.08)
G1 Bosentan 2 mg/kg ^d^	0.28 (−0.32, 0.88)	−54.22 (−197.97, 89.53)	**0.07 (0.03, 0.12)**
G2 Bosentan 4–6 mg/kg	0.07 (−0.12, 0.26)	1.07 (−1.13, 3.26)	0.02 (−0.02, 0.07)
G2 Ciclosporin 5 mg/kg	0.83 ** (0.70, 0.97)	3.78 ** (2.16, 5.4)	0.09 ** (0.06, 0.11)
G2 Rifampicin 2 mg/kg	0.64 ** (0.56, 0.71)	7.20 * (4.49, 9.91)	0.07 ** (0.05, 0.10)

** *p* < 0.01; * *p* < 0.05; ^a^ positive value indicates a decrease in the parameter compared with control; ^b^ letters indicate the site of the study, as detailed in [25]; ^c^ subject 1 excluded from calculations due to issues with data quality during treatment; ^d^ subjects 3, 4, and 6 excluded from calculations due to computational fitting errors.

## Data Availability

Data and code presented in this study are openly available in Zenodo, as follows: 1. DCE-MRI datasets and results in CSV format: https://zenodo.org/record/7506968#.Y7cUhNXP1PY; https://doi.org/10.5281/zenodo.7506968 (published on 5 January 2023). 2. Tracer-kinetic model source code archived from GitHub: https://zenodo.org/record/7507642#.Y7cVF9XP1PZ; https://doi.org/10.5281/zenodo.7507642 (published on 5 January 2023). 3. PMI (Platform for research in Medical Imaging) v3.1 software: https://zenodo.org/record/4382479#.Y7cVntXP1PY; https://doi.org/10.5281/zenodo.4382479 (published on 21 December 2020).

## Supplementary Materials

The following supporting information can be downloaded at: https://www.mdpi.com/article/10.3390/pharmaceutics15030896/s1. Supplementary Sections S1–S6. References [74,75,76,77,78,79,80,81,82,83,84,85,86,87,88,89,90,91,92,93,94,95,96,97,98,99,100,101,102,103,104,105,106,107,108,109,110] are cited in Supplementary Materials.
