# Supplementary material for: Use of In Vivo Imaging and Physiologically-Based Kinetic Modelling to Predict Hepatic Transporter Mediated Drug–Drug Interactions in Rats
Source: Pharmaceutics. 2023 Mar 10;15(3):896. doi: 10.3390/pharmaceutics15030896 (PMC10057977; doi:10.3390/pharmaceutics15030896)
Supplement: Supplementary file 1 [file pharmaceutics-15-00896-s001.zip › pharmaceutics-2184267-supplementary.pdf]

Supplementary Materials

Use of In Vivo Imaging and Physiologically-Based Kinetic Modelling to Predict Hepatic Transporter Mediated Drug–Drug Interactions in Rats

Nicola Melillo [1,2,†], Daniel Scotcher [1,†], J. Gerry Kenna [3], Claudia Green [4], Catherine D. G. Hines [5], Iina Laitinen [6,7], Paul D. Hockings [7,8], Kayode Ogungbenro [1], Ebony R. Gunwhy [9], Steven Sourbron [9], John C. Waterton [3,10], Gunnar Schuetz [4] and Aleksandra Galetin [1,*]

1 Centre for Applied Pharmacokinetic Research, Division of Pharmacy and Optometry, School of Health Science, The University of Manchester, Manchester M13 9PL, UK; daniel.scotcher@manchester.ac.uk (D.S.)
2 SystemsForecastingUK Ltd., Lancaster LA1 5DD, UK
3 Bioxydyn Ltd., Manchester M15 6SZ, UK
4 MR & CT Contrast Media Research, Bayer AG, 13353 Berlin, Germany
5 GSK, Collegeville, PA 19426, USA
6 Sanofi-Aventis Deutschland GmbH, Bioimaging Germany, 65929 Frankfurt am Main, Germany
7 Antaros Medical, 431 83 Mölndal, Sweden
8 MedTech West, Chalmers University of Technology, 413 45 Gothenburg, Sweden
9 Department of Infection, Immunity and Cardiovascular Disease, University of Sheffield, Sheffield S10 2TA, UK
10 Centre for Imaging Sciences, Division of Informatics Imaging & Data Sciences, School of Health Sciences, The University of Manchester, Manchester M13 9PL, UK
* Correspondence: aleksandra.galetin@manchester.ac.uk
† These authors contributed equally to this work.

S1. Pharmacokinetic models for test compounds

Pharmacokinetic models were developed for selected test compounds to inform dose selection in the dynamic contrast enhanced magnetic resonance imaging (DCE-MRI) studies, as described below. The one-compartment (Eq. S1–Eq. S2), two-compartment (Eq. S3–Eq. S5), or three-compartment pharmacokinetic model (Eq. S6–Eq. S9), following intravenous (IV) and oral administration, was fitted to available pharmacokinetic data in rat (**Table S1**). The precision of the parameter estimates (% coefficient of variation; CV) was calculated as described previously [1].

One-compartment model:

$$\frac{dA_G(t)}{dt} = -k_a \cdot A_G(t)$$

$$\frac{dA_1(t)}{dt} = R_{inf} + k_a \cdot A_1(t) - k_{10} \cdot A_1(t) \quad \text{for } 0 < t < T_{inf}$$

$$\frac{dA_1(t)}{dt} = k_a \cdot A_G(t) - k_{10} \cdot A_1(t) \quad \text{for } t > T_{inf} \qquad \text{S1}$$

Copyright: © 2023 by the authors. Licensee MDPI, Basel, Switzerland. This article is an open access article distributed under the terms and conditions of the Creative Commons Attribution (CC BY) license (https://creativecommons.org/licenses/by/4.0/).

$$Y = A_1/V_1 \qquad \text{S2}$$

Two-compartment model:

$$\frac{dA_1(t)}{dt} = -k_a \cdot A_G(t) \qquad \text{S3}$$

$$\frac{dA_1(t)}{dt} = R_{inf} + k_a \cdot A_G(t) - (k_{10} + k_{12})A_1(t) + k_{21} \cdot A_2(t) \quad \text{for } 0 < t < \text{Tinf}$$

$$\frac{dA_1(t)}{dt} = k_a \cdot A_G(t) - (k_{10} + k_{12})A_1(t) + k_{21} \cdot A_2(t) \qquad \text{for } t > \text{Tinf}$$

$$\frac{dA_2(t)}{dt} = k_{12}A_1(t) - k_{21}A_2(t) \qquad \text{S4}$$

$$Y = A_1/V_1 \qquad \text{S5}$$

Three-compartment model:

$$\frac{dA_1(t)}{dt} = R_{inf} - (k_{10} + k_{12} + k_{13})A_1(t) + k_{21} \cdot A_2(t) + k_{31} \cdot A_3(t) \qquad \text{for } 0 < t < \text{Tinf}$$

$$\frac{dA_1(t)}{dt} = -(k_{10} + k_{12} + k_{13})A_1(t) + k_{21} \cdot A_2(t) + k_{31} \cdot A_3(t) \qquad \text{for } t > \text{Tinf} \qquad \text{S6}$$

$$\frac{dA_2(t)}{dt} = k_{12}A_1(t) - k_{21}A_2(t) \qquad \text{S7}$$

$$\frac{dA_3(t)}{dt} = k_{13}A_3(t) - k_{31}A_3(t) \qquad \text{S8}$$

$$Y = A_1/V_1 \qquad \text{S9}$$

Initial conditions for oral dose and IV bolus (R_{inf}=0):

- Oral dose: $A_G(0) = dose \cdot F$, all the other compartments equal to 0;
- IV bolus: $A_1(0) = dose$, all the other compartments equal to 0.

Where t is time, $A_n(t)$ is amount in compartment *n* at time *t*; R_{inf} is IV infusion rate; T_{inf} is the duration of IV infusion; k_a, k_{10}, k_{12}, k_{21}, k_{13} and k_{31} are rate constants; V_1 is volume of compartment 1; F is oral bioavailability; and Y is the measured drug concentration in plasma or blood.

These models assume linear kinetics of the test compounds, despite known non-linearity in pharmacokinetics for some compounds (e.g., ciclosporin, rifampin). The assumption of linear kinetics allowed simplification of the modelling exercise and enabled identification of the model parameters with the available pharmacokinetic data. The models were used to simulate test compound concentrations in plasma under different dosing schedules, to determine suitable doses for in vivo DCE-MRI studies.

Table S1. List of in vivo pharmacokinetic (PK) data in rat used for compartmental model development.

Compound	In vivo PK study design [a]	Reference
Rifampin	20 mg/kg IV	[2]
	20 mg/kg IV	[3]
Asunaprevir	5 mg/kg IV	[4]
Ciclosporin	5.9 mg/kg IV	[5]
	1.7 mg/kg IV	[6]
	3.3 mg/kg IV	[6]
	6.4 mg/kg IV	[6]
Pioglitazone	10 mg/kg PO (Male)	[7]
	10 mg/kg PO (Female)	[7]
	0.5 mg/kg PO	[8]
	5 mg/kg IV	[9]
	10 mg/kg PO	[9]
Bosentan	1 mg/kg IV	[10]
	0.1 mg/kg IV	[11]
	0.3 mg/kg IV	[11]
	1 mg/kg IV	[11]
	3.3 mg/kg IV	[11]
	10 mg/kg IV	[11]
	3.5 mg/kg IV (infusion)	[11]

[a] Intravenous administration is by rapid injection, unless otherwise stated. IV, intravenous; PO, per os (orally).

Simulations were performed to project the inhibition of specific transporters by the test compounds at the specific doses under consideration for in vivo studies, and thereby used to define the expected effects of a single IV dose on gadoxetate DCE-MRI. These simulations used in vitro concentrations causing 50% of maximal transporter inhibition (IC_{50}) or inhibition constant (K_i) data for human and rat hepatobiliary transporters, depending on availability of data (Table 2 and Table 3 in main text). The unbound drug concentration in plasma was used as the driving inhibitory concentration in these simulations; two potential limitations should be noted for this approach. First, increasing evidence supports the "plasma protein-mediated uptake" hypothesis (e.g., [12]), which suggests that the protein-bound drug, as well as the unbound drug in plasma, should be considered available for hepatic uptake by transporters. By extension, it is feasible that the protein-bound drug in plasma may also be relevant for transporter inhibition, although relevant evidence for this is limited. Second, the in vivo liver concentrations of some test compounds are higher than plasma (e.g., ciclosporin [5]), and therefore use of the unbound plasma concentration may lead to under-prediction of transporter inhibition in these simulations. Physiologically-based pharmacokinetic (PBPK) modelling of the test compounds may enable projection of the unbound liver concentration, but this was considered out-of-scope for the current study.

Model fitting and simulations were performed in Matlab (Mathworks, Natick, MA). When needed, pharmacokinetics data from published literature were digitized using GetData Graph Digitizer (http://getdata-graph-digitizer.com/).

S1.1. Rifampin

Rifampin is a well-known, clinically used, inhibitor of organic anion transporting polypeptide 1B1 (OATP1B1; *SLCO1B1*) when administered as single dose, [13,14]. When administered therapeutically to humans, rifampin has been reported to cause both relatively frequent asymptomatic plasma bilirubin elevations and rare symptomatic cholestatic liver injury [15]. The interaction effect of rifampin on gadoxetate DCE-MRI has been previously studied in rat, including for a 10 mg/kg rifampin IV dose in a previous study by the TRISTAN consortium [1]. The 10 mg/kg IV dose of rifampin caused extensive reduction in the gadoxetate-dependent delta R1 in liver, compared to control animals that received saline instead of rifampin. Further, another recent study reported a dose-dependent response (between 1 mg/kg and 20 mg/kg rifampin) of the gadoxetate DCE-MRI relative enhancement profile in liver following administration of rifampin [16]. Thus, the effect of a lower IV dose of rifampin on gadoxetate DCI-MRI was also evaluated in the current study. Based on reported data by Karageorgis et al., (2018), a 2mg/kg dose of rifampin was selected to achieve a moderate effect of rifampin on gadoxetate DCE-MRI profiles in liver. Pharmacokinetic data for rifampin in rat after 2 mg/kg IV dose were not available. A one-compartment pharmacokinetic model for rifampin was developed using published in vivo data in rat [2,3]. Model parameter estimates are presented in **Table S2**. Simulations performed using the model suggested that the plasma concentration of rifampin at 1 h after rifampin IV infusion is expected to be between 2μM and 3μM (**Figure S1**). Such concentrations are slightly lower than typical rifampin maximum plasma concentration (Cmax) measured in clinical studies (4–20 μM; [17]). The pharmacokinetic model for rifampin was developed using data generated following a 20 mg/kg dose in rat [2,3], which is 10-fold higher than the simulated dose (2 mg/kg). In addition, rifampin has non-linear pharmacokinetics. Therefore, the model-based projections for rifampin concentrations in rat plasma must be treated with caution.

Table S2. Parameter estimates and precision for one-compartment model for rifampin in rat.

Parameter	Value	Precision (%CV)
k_{10} (1/h)	0.294	10%
V_1 (mL)	182.1	4%

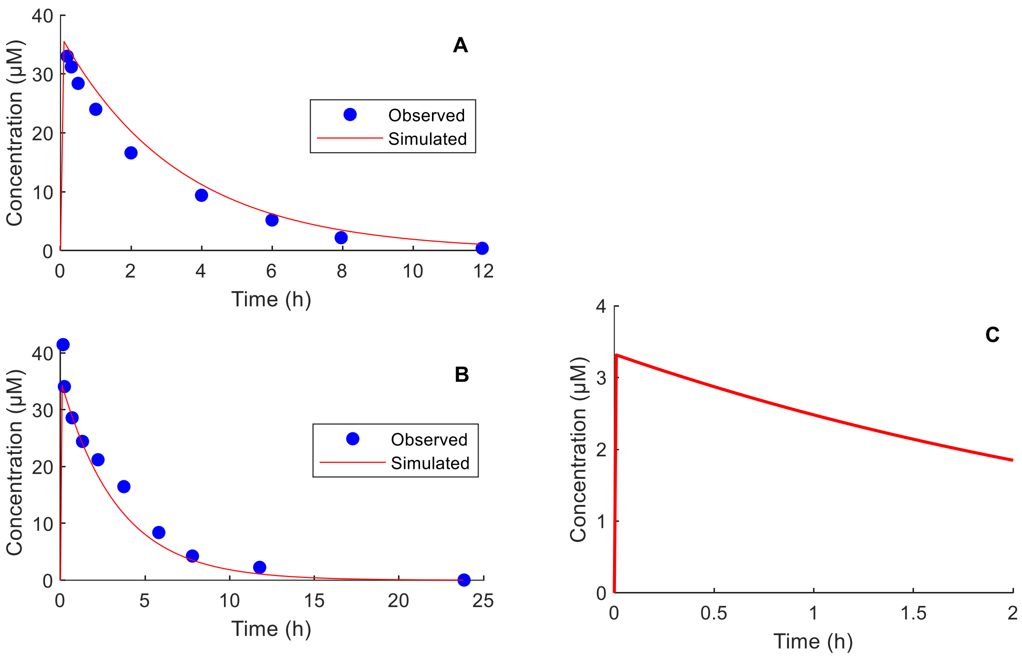

Figure S1. Simulated plasma concentration-time profiles of rifampin in rat after intravenous administration using a two-compartment pharmacokinetic model. The model was developed by fitting the model to pharmacokinetics data from two studies, following single dose, IV administration of 20 mg/kg rifampin Panel A: [2], Panel B: [3]. The developed model was used to simulate rifampin plasma-concentration in rat after IV administration of 2 mg/kg rifampin (Panel C).

S1.2. Asunaprevir

Asunaprevir is an inhibitor of hepatic OATP1B transporters in vitro (**Table 3** in main text), and caused a modest drug–drug interaction with OATP1B substrate rosuvastatin (ratio of area under the plasma concentration-time curve (AUC) between the presence and absence of inhibitor (AUCR) < 2-fold; [18]. In patients with hepatitis C virus infection, administration of asunaprevir in combination with daclatasvir has been reported to cause rare liver injury, liver injury, cholecystitis, and disseminated intravascular coagulation with a fever and skin rash, which is suggestive of an immunoallergic mechanism [19].

Asunaprevir dosing for the gadoxetate DCE-MRI study in rat was established using pharmacokinetic modelling, to find a dose that would achieve the primary aim of obtaining clinically relevant total drug concentrations of asunaprevir in rat plasma at the time of initiating gadoxetate DCE-MRI. The model was also used to project the in vivo inhibition of the hepatic Oatp1 transporter in rat at the selected dose, based upon in vitro OATP1B1 IC_{50} data for asunaprevir (Eq. S10)

$$Fraction\ transporter\ activity\ remaining = \frac{1}{1+\frac{Cp \cdot fu}{IC_{50}}} \qquad S10$$

A two-compartment pharmacokinetic model (Table S3) was developed for asunaprevir, through fitting to published plasma concentration-time data in rat, following IV administration of 5 mg/kg asunaprevir [4]. Model simulations projected that 0.5 h after a 5 mg/kg IV dose, the plasma concentration of asunaprevir would be within the clinically relevant range (**Figure S2**, Panels A and B). Further, 0.5 h after administration of 5 mg/kg asunaprevir, the simulations project a minor inhibition of hepatobiliary transporters based on IC_{50} data for the human transporters (**Figure S2**, Panel C), while modest inhibition of rat Na+-taurocholate cotransporting polypeptide (rNtcp; *Slc10a1*) was simulated (**Figure S2**, Panel D).

Table S3. Parameter estimates and precision (% coefficient of variation; CV) for two-compartment model [a] for asunaprevir in rat.

Parameter	Value	Precision (%CV)
k_{10} (1/h)	6.79	19%
k_{12} (1/h)	3.71	27%
k_{21} (1/h)	1.60	34%
V_1 (mL)	96.0	17%

[a] See Eq. S3–Eq. S5

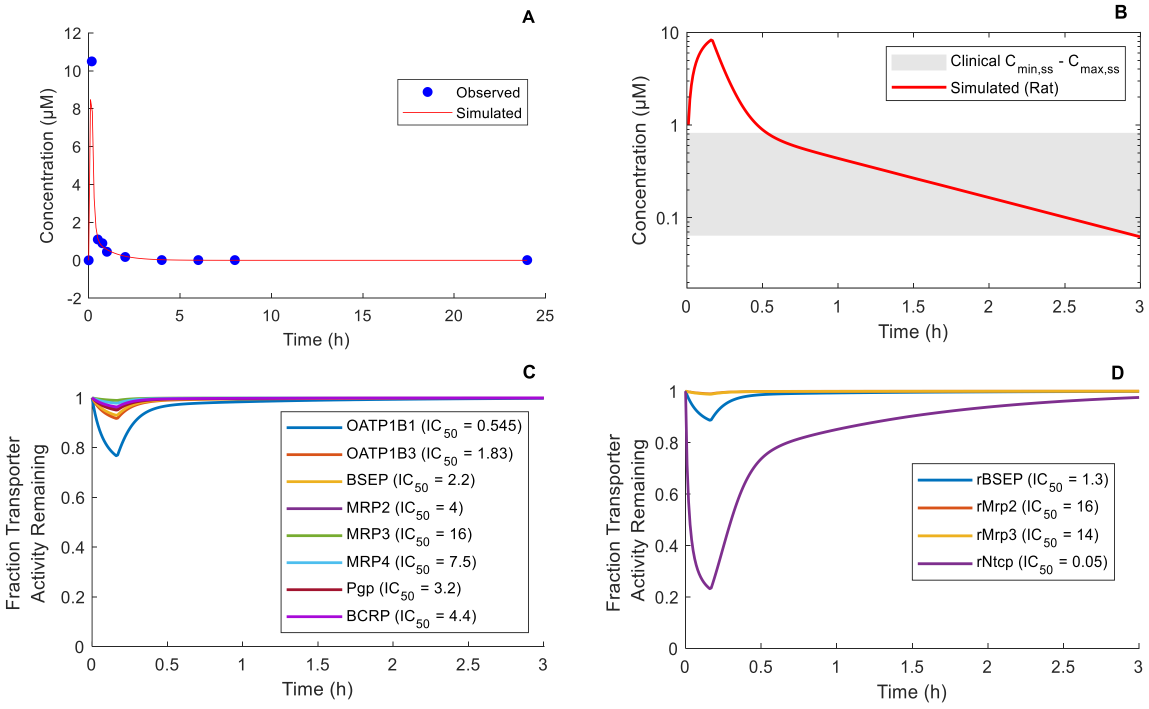

Figure S2. Simulated plasma concentration-time profiles of asunaprevir and projected transporter inhibition in rat after intravenous administration using a two-compartment pharmacokinetic model. Panel A: Model fit to pharmacokinetics data following single dose, IV administration of 5 mg/kg asunaprevir [4]. Panel B: Simulated asunaprevir plasma concentration in rat after 5 mg/kg dose in comparison to clinical steady-state drug concentrations [20]. Panel C: Projected fraction of transporter activity remaining-time curves following asunaprevir 5mg/kg IV dose in rat, based on human transporter IC_{50} data. Panel D: Projected fraction of transporter activity remaining-time curves following asunaprevir 5 mg/kg IV dose in rat, based on rat transporter IC_{50} data.

S1.3. Ciclosporin

Ciclosporin is an inhibitor of hepatic transporters, including OATP1B and multidrug resistance-associated protein (MRP)2 (*ABCC2*), in vitro (**Table 2 and 3** in main text); OATP1B1 inhibition is considered to mediate the clinical drug-drug interaction between ciclosporin and pitavastatin [21]. Ciclosporin administration to patients may cause dose-related hyperbilirubinaemia, which commonly is asymptomatic and reversible on dose reduction [22].

Ciclosporin exhibits non-linear binding to erythrocytes in blood [23], with pharmacokinetics data typically reported as blood concentration data, rather than plasma concentration data. Therefore, a pharmacokinetic model for ciclosporin was developed using blood concentration measurements in rat following IV administration (**Figure S3**, **Table S4**). Subsequently, unbound concentrations of ciclosporin were calculated as previously reported (Eq. S11) [23].

$$\boldsymbol{C_u = 0.5 \cdot \Big[C_B - \{(1 - Hct)\cdot fu + Hct\}\cdot K_D - Hct \cdot nP_T + \{(C_B - \{(1 - Hct)\cdot fu + Hct \cdot K_D - Hct\cdot nP_T)^2 + 4 \cdot \{(1 - Hct)\cdot fu + Hct\}\cdot K_D \cdot C_B\}^{\frac{1}{2}} \Big] / \{(1 - Hct)\cdot fu + Hct\}} \quad \text{S11}$$

Where C_u, C_B, Hct, fu, K_D, and nP_T are the unbound concentration, blood concentration, haematocrit, fraction unbound in plasma, erythrocyte dissociation constant (0.158 µg/ mL), and binding capacity (4.64 µg·Eq/mL), respectively.

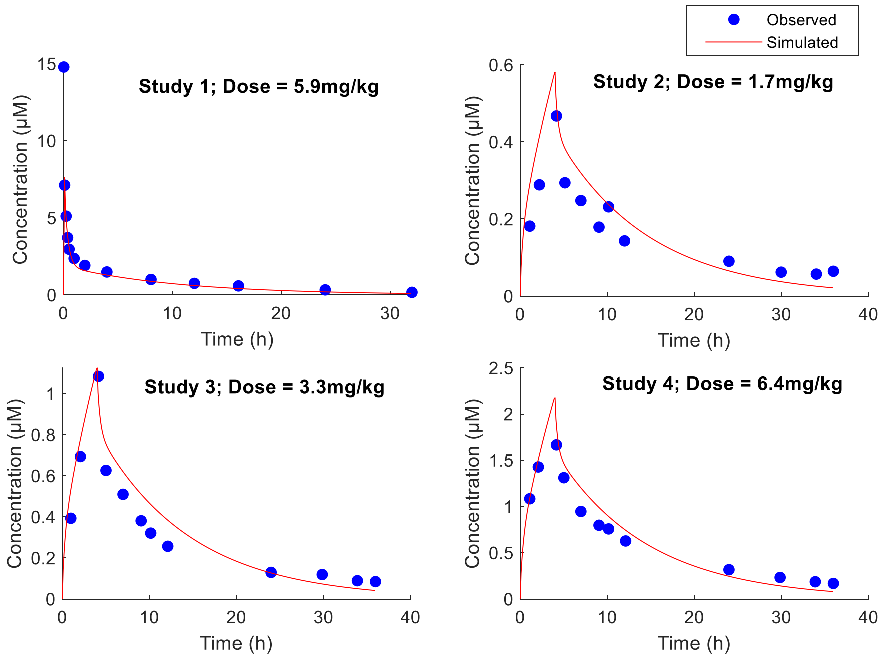

Figure S3. Simulated blood concentration-time profiles of ciclosporin using a three-compartment pharmacokinetic model, compared with the in vivo data used for model fitting. The observed data were obtained following IV administration of ciclosporin. Study 1: [5]; studies 2, 3 and 4: [6].

Table S4. Parameter estimates and precision (% coefficient of variation; CV) for three-compartment model [a] for ciclosporin in rat.

Parameter	Value	Precision (%CV)
k_{10} (1/h)	0.881	6%
k_{12} (1/h)	13.8	17%
k_{21} (1/h)	14.6	16%
k_{13} (1/h)	4.64	7%
K_{31} (1/h)	0.715	9%
V_1 (mL)	63.5	3%

[a] See Eq. S6–Eq. S9

Simulations projected that the blood concentration of ciclosporin in rat would be comparable to clinically reported blood concentrations one hour after 5 mg/kg IV administration of ciclosporin (**Figure S4**, Panel A). Projections of the in vivo inhibition effects of ciclosporin accounted for the wide variability in reported in vitro transporter inhibition data in literature sources, in particular considering the effects of pre-incubation [24]. At one hour after ciclosporin administration, > 75% inhibition of OATP1B transporters was predicted, while minimal inhibition of MRP2 was predicted.

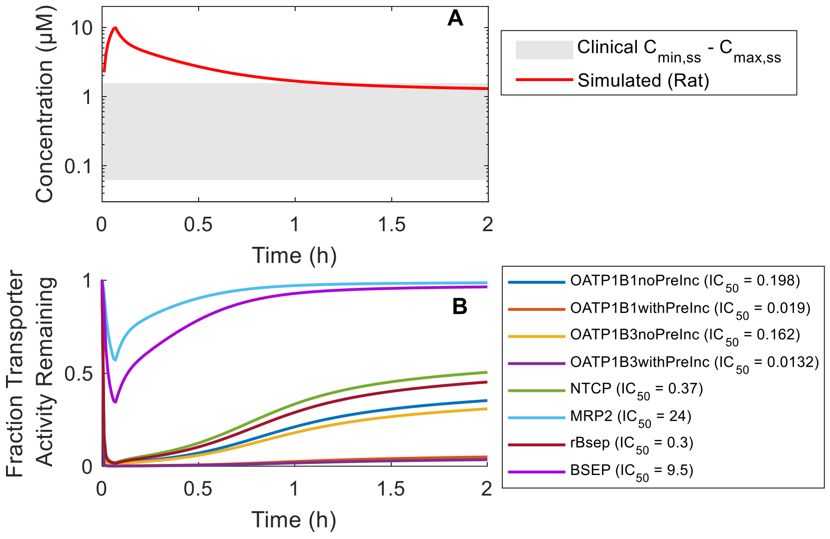

Figure S4. Simulated plasma concentration-time profiles of ciclosporin and projected transporter inhibition in rat after intravenous administration using a three-compartment pharmacokinetic model. Panel A: Simulated ciclosporin blood concentration in rat after 5mg/kg dose in comparison to clinical steady-state drug concentrations [25]. Panel B: Projected fraction of transporter activity remaining-time curves following ciclosporin 5mg/kg IV dose in rat, based on transporter IC_{50} data.

S1.4. Ketoconazole

Ketoconazole has well-reported hepatotoxicity, which typically is of hepatocellular pattern, and rarely is associated with cholestasis; the most frequent consequence is mild and reversible asymptomatic liver injury, although severe liver damage may arise rarely [26]. Although ketoconazole can be shown to inhibit OATP1B in vitro (**Table 3** in main text), such inhibition is unlikely to cause drug–drug interactions in the clinical setting [27]. Ketoconazole also did not inhibit MRP2 in vitro. The pharmacokinetics of ketoconazole in rat after IV administration was reported previously [28], and indicated that 30 min after administration of 3 mg/kg ketoconazole, the plasma concentration (4 μM) was within the clinical Cmax (7 μM) and Ctrough (0.2 μM) [29].

S1.5. Pioglitazone

Pioglitazone is a bile salt export pump (BSEP; *ABCB11*) inhibitor in vitro, which has been reported to cause extremely infrequent hepatocellular-cholestatic drug-induced liver injury (DILI) [30]. Pioglitazone is considered to pose markedly less clinical DILI concern than many other BSEP inhibitors, such as troglitazone and bosentan [31]. In vivo

pharmacokinetics data in rat for pioglitazone are limited. Therefore, pharmacokinetics data following oral administration was included in the model development for pioglitazone, to support parameter estimation. A one-compartment pharmacokinetic model was developed (**Figure S5** and **Table S5**). Subsequently, simulations were performed at various doses to establish a dose that would achieve clinical plasma exposure in rat for the DCE-MRI study (**Figure S6**, Panels A and C). Based on these simulations, the 0.4 mg/kg IV dose, administered 30 min prior to DCE-MRI, was selected for the current in vivo experiments. Simulations suggest that minimal inhibition of OATP1B and other transporters would be expected at the selected 0.4 mg/ kg dose (**Figure S6**, Panels B and D).

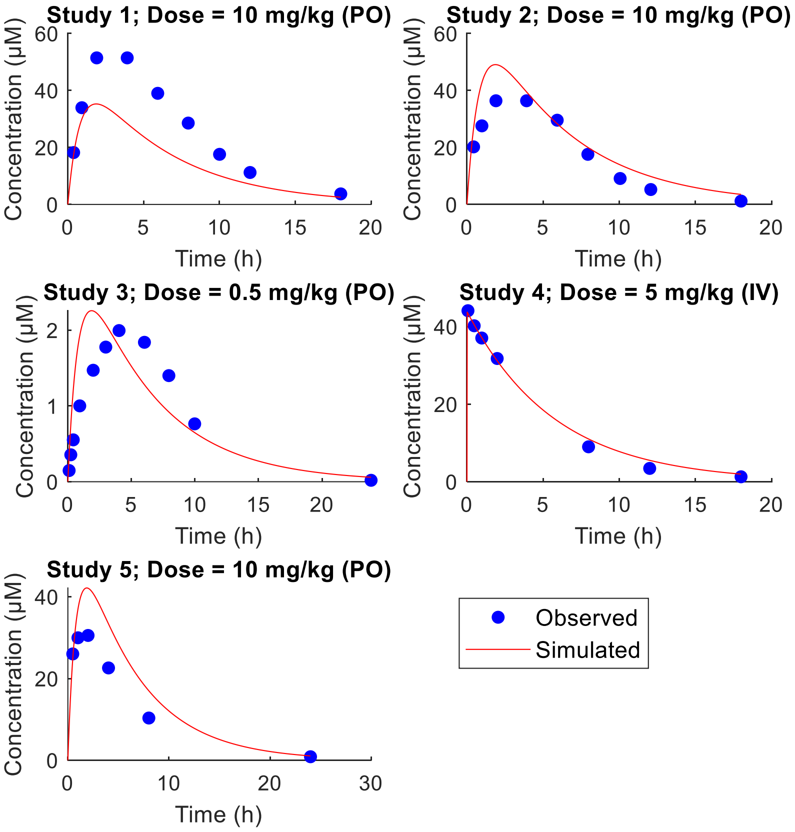

Figure S5. Simulated blood concentration-time profiles of pioglitazone using a one-compartment pharmacokinetic model (with depot compartment for oral administration), compared with the in vivo data used for model fitting. The observed data were obtained following IV or oral administration of pioglitazone. Studies 1 and 2: [7]; study 3 [8]; studies 4 and 5: [9].

Table S5. Parameter estimates and precision (% coefficient of variation; CV) for two-compartment model [a] for pioglitazone in rats.

Parameter	Value	Precision (%CV)
k_a (1/h)	1.2	39%
F	0.66	19%
k_{10} (1/h)	0.17	26%
V_1 (mL)	88	13%

[a] See Eq. S3–Eq. S5

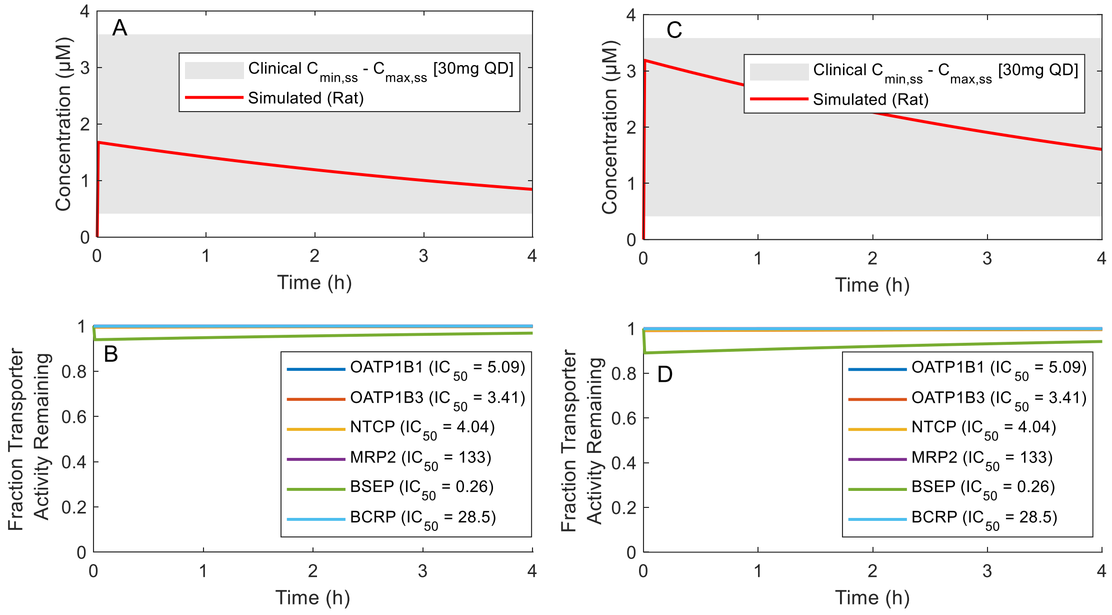

Figure S6. Simulated plasma concentration-time profiles of pioglitazone (Panel A and C) and projected transporter inhibition (Panel C and D) in rat after intravenous administration, using a one-compartment pharmacokinetic model. Panels A and B show simulations for a 0.21 mg/kg IV dose of pioglitazone; Panels C and D show simulations for a 0.4 mg/kg IV dose of pioglitazone. The grey shaded areas in Panels A and C represent the clinical steady state trough ($C_{min,ss}$) and maximum ($C_{max,ss}$) concentrations of pioglitazone for a 30 mg QD dosing of pioglitazone [32].

S1.6. Bosentan

Bosentan is associated with mild liver dysfunction in about 10% of treated patients, and also with rare, severe hepatotoxicity, which may result in liver failure [33]; indeed, the USFDA approved prescribing information includes a so-called black boxed warning for hepatotoxicity [34]. Two- and three-compartment pharmacokinetic models were evaluated to describe the in vivo pharmacokinetics data for bosentan in rat (**Figure S7** and **Figure S8**, and **Table S6** and **Table S7**). The three-compartment model could achieve a fitting that appeared better than the two-compartment model, based on visual check. Conversely, estimates of the k_{13} and k_{31} rate constants for the three-compartment model had poor precision (**Table S7**), and the parameter estimates of the three-compartment model were highly sensitive to initial estimates (not shown). Therefore, the two-compartment model was selected as being more reliable for subsequent simulations. Simulations using the two-compartment model were performed at various doses, to establish a dose that would achieve clinical plasma exposure in rat for the DCE-MRI study (Figure S9, Panel A). A 2 mg/kg IV dose, administered 60 min prior to DCE-MRI, was selected for the current in vivo experiments. At this dose, minimal OATP1B inhibition was projected by the simulations (**Figure S9**, Panel B).

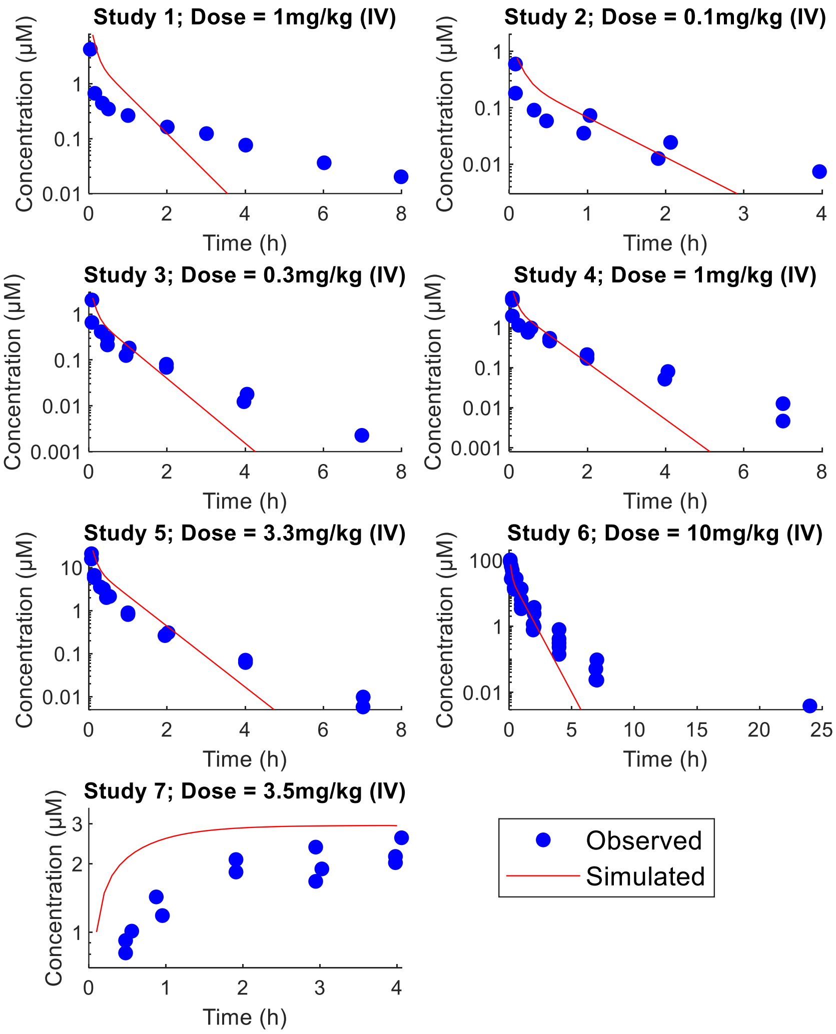

Figure S7. Simulated plasma concentration-time profiles of bosentan using a two-compartment pharmacokinetic model, compared with the in vivo data used for model fitting. The observed data were obtained following IV administration of bosentan. Study 1: [10]; studies 2 to 7: [11].

Table S6. Parameter estimates and precision (% coefficient of variation; CV) for two-compartment model [a] for bosentan in rats.

Parameter	Value	Precision (%CV)
k_{10} (1/h)	5.0	11%
k_{12} (1/h)	3.8	45%
k_{21} (1/h)	3.5	48%
V_1 (mL)	32	13%

[a] See Eq. S3–Eq. S5

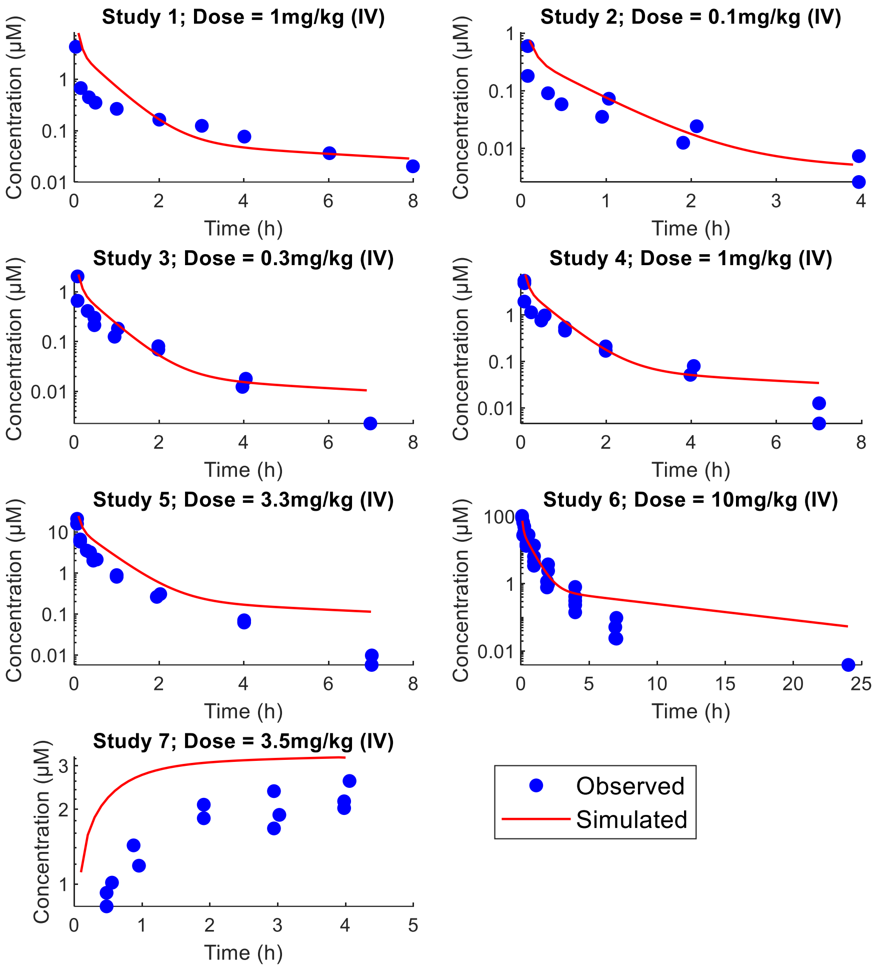

Figure S8. Example simulated plasma concentration-time profiles of bosentan using a three-compartment pharmacokinetic model and parameter estimates listed in Table S7, compared with the in vivo data used for model fitting. The observed data were obtained following IV administration of bosentan. Study 1: [10]; studies 2 to 7: [11].

Table S7. Example [a] parameter estimates and precision (% coefficient of variation; CV) for three-compartment model [b] for bosentan in rats.

Parameter	Value	Precision (%CV)
k_{10} (1/h)	5.1	31%
k_{12} (1/h)	6.1	34%
k_{21} (1/h)	4.3	43%
k_{13} (1/h)	0.90	78%
K_{31} (1/h)	0.13	560%
V_1 (mL)	26	20%

[a] Parameter estimates were highly sensitive to initial estimates; [b] see Eq. S6–Eq. S9

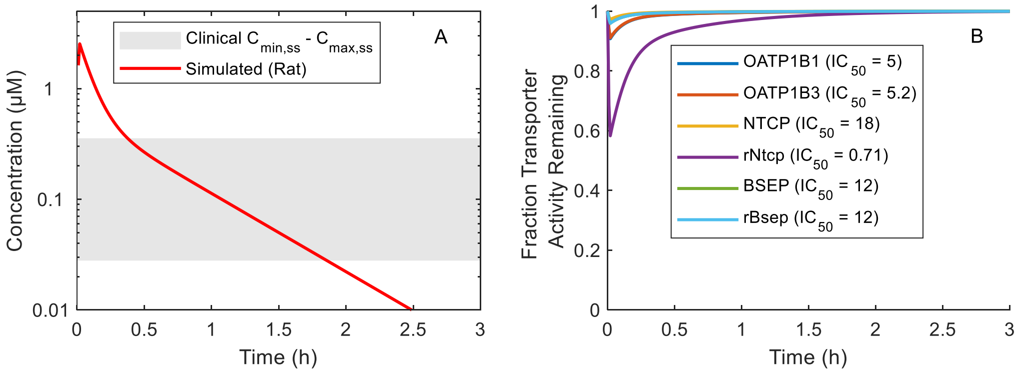

Figure S9. Simulated plasma concentration-time profiles of bosentan (Panel A) and projected transporter inhibition (Panel B) in rat after 2 mg/kg intravenous administration, using a two-compartment pharmacokinetic model. The grey shaded area in Panel A represents the clinical steady state trough ($C_{min,ss}$) and maximum ($C_{max,ss}$) concentrations.

S2. Preparation of test compounds for intravenous injection

Table S8. Formulation of test compounds for intravenous injection

Test compound (dose)	Preparation
Rifampicin (2 mg/kg)	Eremfat® 300 mg tablet dissolved in 5 mL water-for-injection (WFI) to produce a 60 mg/mL solution. Dilute the 60 mg/mL solution 1:30 in saline to produce a 2 mg/mL solution. Inject 1 mL of 2 mg/mL solution per kg bodyweight
Asunaprevir (5 mg/kg)	Asunaprevir dissolved in 9:1 PEG-400/ethanol solution for injection.
Bosentan (2 and 4–6 mg/kg)	Bosentan 1 mg dissolved in 50 μL of 95% ethanol, then add 20–50 μL of Tween80 and add WFI to a total volume of 1 mL (1 mg/mL solution). Inject 2 mL of 1 mg/mL per kg bodyweight.
Ciclosporin (5 mg/kg)	Sandimmune, Novartis, (50 mg/mL) diluted 1:10 in saline. Inject 1 mL of 5 mg/mL solution per kg bodyweight.
Ketoconazole (3 mg/kg)	Ketoconazole HRA 200 mg tablet crushed to fine powder and subsequently dissolved in 40 mL of 9:1 PEG400:propylene glycol solution to produce a 5 mg/mL solution. Inject 0.6 mL of 5 mg/mL solution per kg bodyweight.
Pioglitazone (0.4 mg/kg)	Pioglitazone dissolved in 5% ethanol/ 70% (10%) 2-hydroxypropyl-β-cyclodextrin (HPCD)/ 25% saline for injection.

S3. Representative MRI images showing region of interest selection

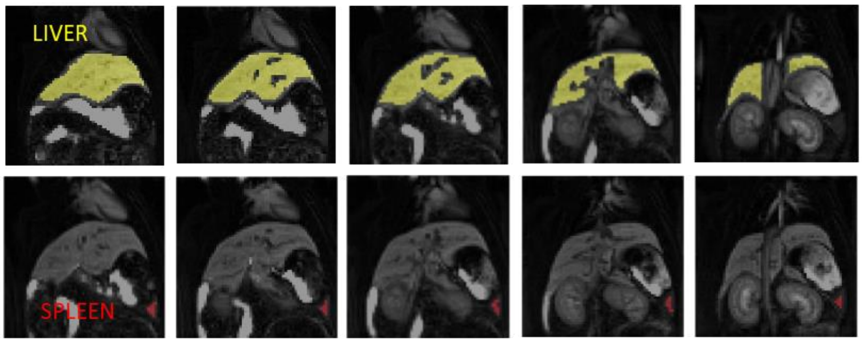

Figure S10. Representative series of gadoxetate dynamic contrast enhanced (DCE) images, showing manually selected regions of interest, covering liver (yellow, top panels) and spleen (red, bottom panels) in rat.

S4. Alternative modelling assumptions for inhibition of hepatobiliary efflux in PBPK model

The contribution of the inhibition of efflux transporters to drug–drug interactions is challenging to understand, with limited previous efforts in the literature. In particular, the in vivo consequences of such inhibition are challenging to delineate, even when typical in vivo pharmacokinetics data are available (e.g., [35]). As such, there is a lack of consensus of best practice on this topic. One challenge is the assumption of the operational concentration to drive the inhibition of efflux transporters within PBPK modelling framework. Assumptions of this operational concentration of perpetrator could include the unbound plasma concentration (e.g., [36]) or the unbound intracellular concentration (e.g., [37,38]). Here, PBPK predictions of test compound effects on gadoxetate DCE-MRI are performed, with alternative assumptions of (a) no inhibition of hepatobiliary efflux by the test compounds, and (b) inhibition of hepatobiliary efflux is driven by the unbound concentration of the test compound in the hepatocyte. In the case of (b), the intracellular concentration was modelled using the unbound concentration ratio between hepatocyte and plasma of the test compound (Kpuu; **Table S9**), from literature and the pharmacokinetic models in Section S1 (above).

$$CL_{transporter,inh} = \frac{CL_{transporter}}{1 + c_{u,p}(t) \times Kpuu/IC_{50}} \quad \text{S12}$$

Table S9. Kpuu data for test compounds, collated from literature

Test compound	Kpuu	Reference
Rifampicin	15.9	[39]
Asunaprevir	11.5	[40]
Bosentan	3	[41]
Ciclosporin	17	[41]
Ketoconazole	1	[41]
Pioglitazone	No data found	-

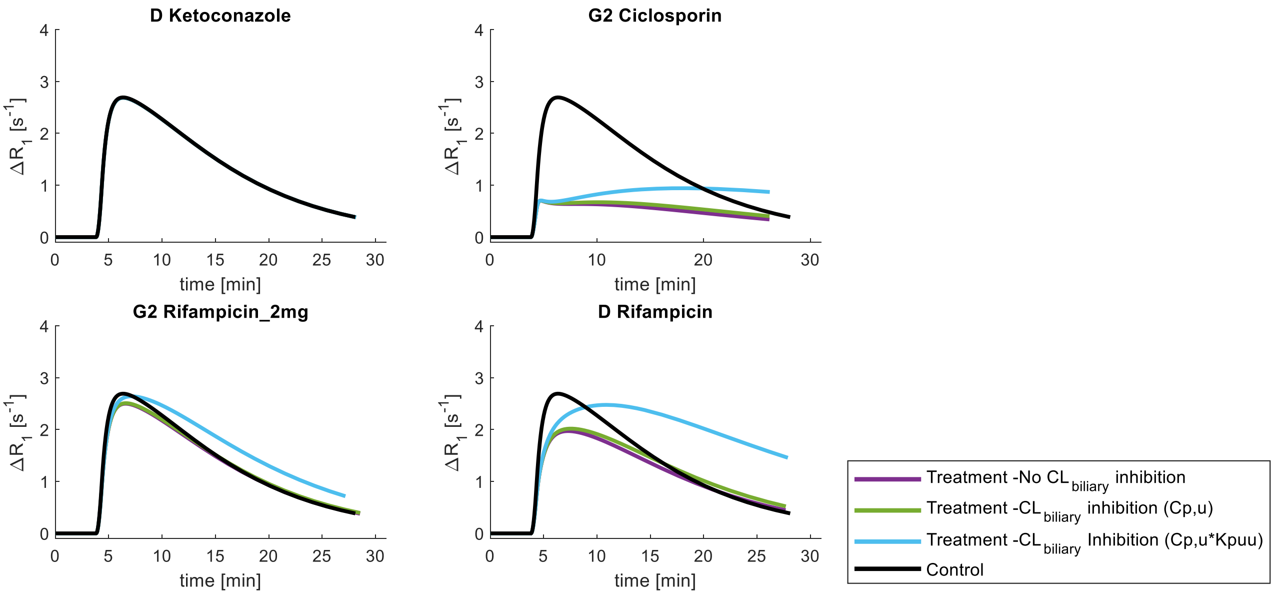

Figure S11. Impact of different assumptions of test compound inhibition of hepatobiliary efflux transport ($CL_{biliary}$) on simulated gadoxetate DCE-MRI profiles in liver for selected test compounds. Simulations of the control phase (absence of test compound) are also presented for reference.

S5. Modelling of input function for tracer-kinetic (TK) model

The tracer-kinetic model takes the time-dependent plasma concentration of gadoxetate (cp(t)) as input function (see Equation 6 in main text). Considering the difficulty in reliably measuring cp(t) in small animals, the TK model used in this study implemented a standardized cp(t), derived from a simplified, two-compartment model of the rat circulation, as shown in **Figure S12**.Within this model, literature values for rats are used for all constants, as shown in **Table S10**. In **Figure S13**, observed gadoxetate time profiles in rat livers are shown against profiles fitted using the TK model with this standardized cp(t) incorporated.

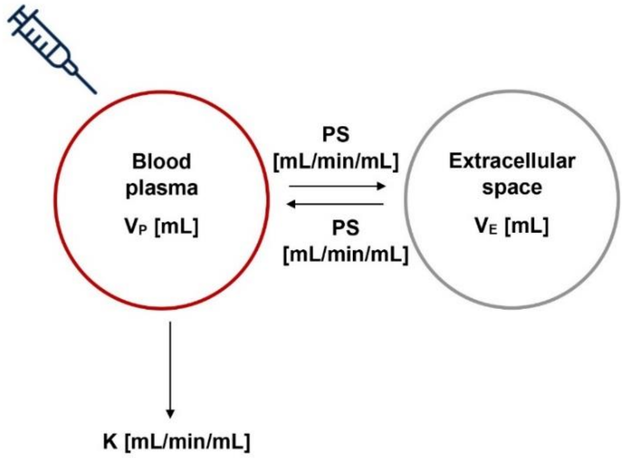

Figure S12. Tracer-kinetic model for blood concentrations in rats. PS = whole body permeability-surface for gadoxetate; V_P = whole body plasma volume; V_E = whole body extracellular volume; K = GFR + (K^{trans})(V_L), where GFR is the glomerular filtration rate, K^{trans} is the hepatic plasma clearance rate, and V_L is the liver volume.

Table S10. Sources of literature values used for the simplified model of rat circulation shown in **Figure S12**

Variable	Value	Reference
PS	0.172 mL^{-S}	Estimated from study data using the PBPK model from Table 3 in [1]
Haematocrit, Hct	0.418	[42]
Whole body blood volume, V_B	15.8 mL	When assuming a body weight of 250 g [43]
Whole body extracellular volume, V_E	30 mL	[1]
Glomerular filtration rate, GFR	0.023 mL^{-S}	[44]
Liver volume, V_L	8.47 mL	[1]

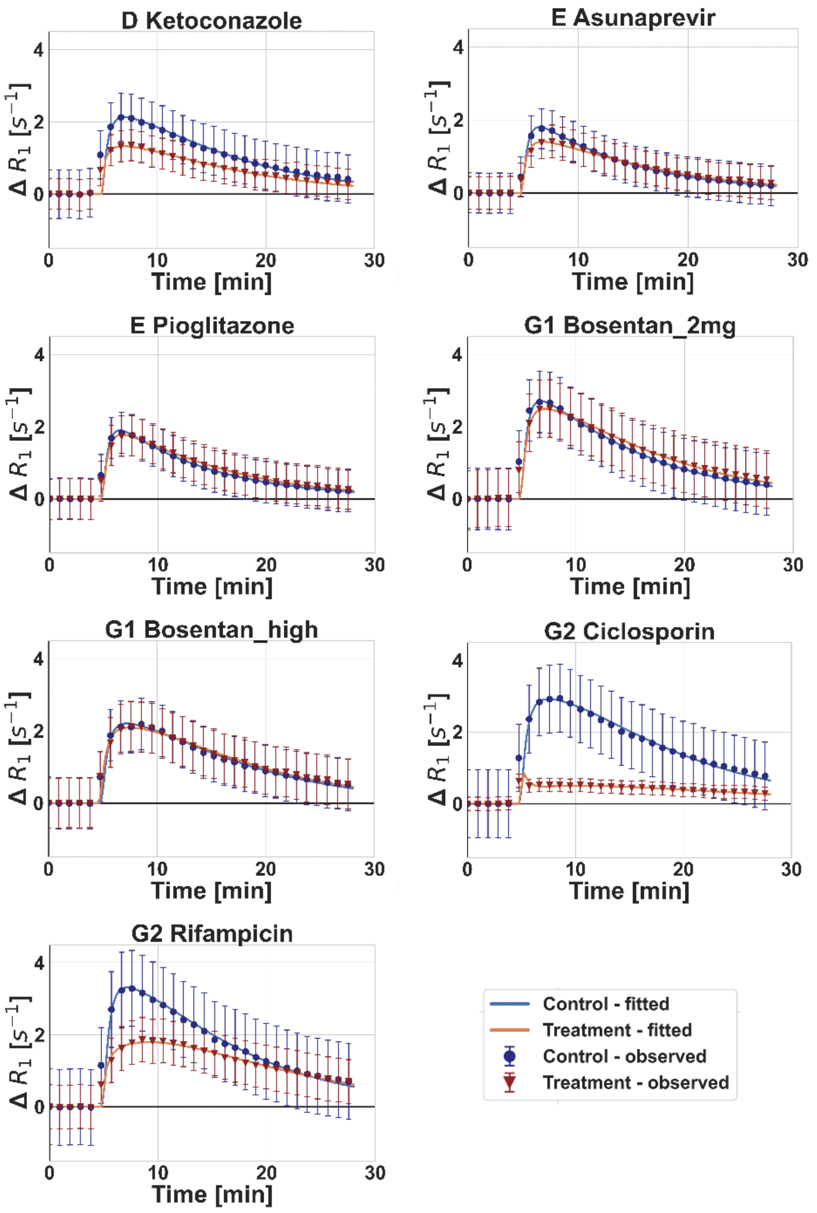

Figure S13. Fitted versus observed liver ΔR_1-time profiles for different sites (D, E, G1, and G2) and perpetrators (asunaprevir, bosentan, ciclosporin, ketoconazole, pioglitazone, and rifampicin). The tracer-kinetic model was fitted to the mean observed liver profiles, using a standardised $c_a(t)$ derived from a simplified model of rat circulation as input function. Observed data represent mean average profiles over all rats per site, with the error bars representing the standard deviation.

S6. Comparison of transport parameters between PBPK and tracer-kinetic model

Table S11. Comparison of the definition, units, and values of gadoxetate transport parameters between the physiologically-based pharmacokinetic model and the tracer-kinetic model, in the original form used by each model, and a 'unified' set of units/definition that allows direct comparison of the values.

**Physiological process**	**Hepatic uptake**		**Hepatobiliary efflux**	
**Model**	**PBPK**	**TK**	**PBPK**	**TK**
**Parameter**	CL_{uptake}	k_{he}	$CL_{biliary}$	k_{bh}
**Original units**	*L/ h*	*mL/ min/ mL tissue volume*	*L/ h*	*mL/ min/ mL tissue volume*
**Original reference concentration**	*Unbound concentration in liver interstitial fluid*	*Total concentration in liver interstitial fluid*	*Unbound concentration in the hepatocytes*	*Total concentration in hepatocytes*
**Original value**	2.17 [a]	1.31[b]	0.07 [a]	0.14[b]
**Unified units** [c]	*mL/min*		*mL/min*	
**Unified reference concentration**	*Total concentration in the liver interstitial fluid*		*Total concentration in hepatocytes*	
**New value**	36.2	11.1	0.756	1.19
**Fold difference** [d]	3.3-fold		0.63-fold	

[a] Table 3 in [1]; [b] mean of all animals in the current study in absence of test compound; [c] assumptions: (i) liver volume is 8.47 mL, derived from Table S2 in [1], (ii) gadoxetate has negligible binding to plasma proteins such as albumin [45], (iii) PBPK model assumes fraction unbound in hepatocytes is 0.648 [1]; [d] calculated as $\frac{PBPK}{TK}$. PBPK, physiologically-based pharmacokinetic; TK, tracer-kinetic; CL_{uptake}, active uptake clearance; k_{he} gadoxetate transport from the extracellular space into hepatocytes; k_{bh} gadoxetate transport from hepatocytes into bile

References

1. Scotcher, D.; Melillo, N.; Tadimalla, S.; Darwich, A.S.; Ziemian, S.; Ogungbenro, K.; Schutz, G.; Sourbron, S.; Galetin, A. Physiologically Based Pharmacokinetic Modeling of Transporter-Mediated Hepatic Disposition of Imaging Biomarker Gadoxetate in Rats. *Mol. Pharm.* **2021**, *18*, 2997-3009, doi:10.1021/acs.molpharmaceut.1c00206.
2. Lau, Y.Y.; Okochi, H.; Huang, Y.; Benet, L.Z. Pharmacokinetics of atorvastatin and its hydroxy metabolites in rats and the effects of concomitant rifampicin single doses: relevance of first-pass effect from hepatic uptake transporters, and intestinal and hepatic metabolism. *Drug Metab Dispos* **2006**, *34*, 1175-1181, doi:10.1124/dmd.105.009076.
3. Jiang, R.; Dong, J.; Li, X.; Du, F.; Jia, W.; Xu, F.; Wang, F.; Yang, J.; Niu, W.; Li, C. Molecular mechanisms governing different pharmacokinetics of ginsenosides and potential for ginsenoside-perpetrated herb-drug interactions on OATP1B3. *Br J Pharmacol* **2015**, *172*, 1059-1073, doi:10.1111/bph.12971.
4. Mosure, K.W.; Knipe, J.O.; Browning, M.; Arora, V.; Shu, Y.Z.; Phillip, T.; McPhee, F.; Scola, P.; Balakrishnan, A.; Soars, M.G.; et al. Preclinical Pharmacokinetics and In Vitro Metabolism of Asunaprevir (BMS-650032), a Potent Hepatitis C Virus NS3 Protease Inhibitor. *J. Pharm. Sci.* **2015**, *104*, 2813-2823, doi:10.1002/jps.24356.
5. Kawai, R.; Mathew, D.; Tanaka, C.; Rowland, M. Physiologically based pharmacokinetics of cyclosporine A: extension to tissue distribution kinetics in rats and scale-up to human. *J Pharmacol Exp Ther* **1998**, *287*, 457-468.
6. Lindberg-Freijs, A.; Karlsson, M.O. Dose dependent absorption and linear disposition of cyclosporin A in rat. *Biopharm Drug Dispos* **1994**, *15*, 75-86, doi:10.1002/bdd.2510150107.
7. Fujita, Y.; Yamada, Y.; Kusama, M.; Yamauchi, T.; Kamon, J.; Kadowaki, T.; Iga, T. Sex differences in the pharmacokinetics of pioglitazone in rats. *Comp. Biochem. Physiol. C Toxicol. Pharmacol.* **2003**, *136*, 85-94, doi:10.1016/s1532-0456(03)00194-7.
8. Maeshiba, Y.; Kiyota, Y.; Yamashita, K.; Yoshimura, Y.; Motohashi, M.; Tanayama, S. Disposition of the new antidiabetic agent pioglitazone in rats, dogs, and monkeys. *Arzneimittelforschung.* **1997**, *47*, 29-35.
9. Umathe, S.N.; Dixit, P.V.; Kumar, V.; Bansod, K.U.; Wanjari, M.M. Quercetin pretreatment increases the bioavailability of pioglitazone in rats: involvement of CYP3A inhibition. *Biochem. Pharmacol.* **2008**, *75*, 1670-1676, doi:10.1016/j.bcp.2008.01.010.
10. Treiber, A.; Schneiter, R.; Delahaye, S.; Clozel, M. Inhibition of organic anion transporting polypeptide-mediated hepatic uptake is the major determinant in the pharmacokinetic interaction between bosentan and cyclosporin A in the rat. *J Pharmacol Exp Ther* **2004**, *308*, 1121-1129, doi:10.1124/jpet.103.061614.
11. Li, R.; Niosi, M.; Johnson, N.; Tess, D.A.; Kimoto, E.; Lin, J.; Yang, X.; Riccardi, K.A.; Ryu, S.; El-Kattan, A.F.; et al. A Study on Pharmacokinetics of Bosentan with Systems Modeling, Part 1: Translating Systemic Plasma Concentration to Liver Exposure in Healthy Subjects. *Drug Metab Dispos* **2018**, *46*, 346-356, doi:10.1124/dmd.117.078790.
12. Francis, L.J.; Houston, J.B.; Hallifax, D. Impact of Plasma Protein Binding in Drug Clearance Prediction: A Data Base Analysis of Published Studies and Implications for In Vitro-In Vivo Extrapolation. *Drug Metab Dispos* **2021**, *49*, 188-201, doi:10.1124/dmd.120.000294.
13. Barnett, S.; Ogungbenro, K.; Ménochet, K.; Shen, H.; Humphreys, W.G.; Galetin, A. Comprehensive Evaluation of the Utility of 20 Endogenous Molecules as Biomarkers of OATP1B Inhibition Compared with Rosuvastatin and Coproporphyrin I. *J Pharmacol Exp Ther* **2019**, *368*, 125-135, doi:10.1124/jpet.118.253062.
14. US Food Drug Admin. Drug Development and Drug Interactions: Table of Substrates, Inhibitors and Inducers. Available online: https://www.fda.gov/drugs/drug-interactions-labeling/drug-development-and-drug-interactions-table-substrates-inhibitors-and-inducers (accessed on 18th March 2022).
15. Rifampin. In *LiverTox: Clinical and Research Information on Drug-Induced Liver Injury*; National Institute of Diabetes and Digestive and Kidney Diseases: Bethesda (MD), 2012.
16. Karageorgis, A.; Lenhard, S.C.; Yerby, B.; Forsgren, M.F.; Liachenko, S.; Johansson, E.; Pilling, M.A.; Peterson, R.A.; Yang, X.; Williams, D.P.; et al. A multi-center preclinical study of gadoxetate DCE-MRI in rats as a biomarker of drug induced inhibition of liver transporter function. *PLoS ONE* **2018**, *13*, e0197213, doi:10.1371/journal.pone.0197213.
17. Abulfathi, A.A.; Decloedt, E.H.; Svensson, E.M.; Diacon, A.H.; Donald, P.; Reuter, H. Clinical Pharmacokinetics and Pharmacodynamics of Rifampicin in Human Tuberculosis. *Clin. Pharmacokinet.* **2019**, *58*, 1103-1129, doi:10.1007/s40262-019-00764-2.
18. Eley, T.; Han, Y.H.; Huang, S.P.; He, B.; Li, W.; Bedford, W.; Stonier, M.; Gardiner, D.; Sims, K.; Rodrigues, A.D.; et al. Organic anion transporting polypeptide-mediated transport of, and inhibition by, asunaprevir, an inhibitor of hepatitis C virus NS3 protease. *Clin Pharmacol Ther* **2015**, *97*, 159-166, doi:10.1002/cpt.4.
19. Miyashima, Y.; Honma, Y.; Miyagawa, K.; Oe, S.; Senju, M.; Shibata, M.; Hiura, M.; Abe, S.; Harada, M. Daclatasvir and Asunaprevir Combination Therapy-induced Hepatitis and Cholecystitis with Coagulation Disorder due to Hypersensitivity Reactions. *Intern. Med.* **2016**, *55*, 3595-3601, doi:10.2169/internalmedicine.55.7347.
20. Eley, T.; Sevinsky, H.; Huang, S.P.; He, B.; Zhu, K.; Kandoussi, H.; Gardiner, D.; Grasela, D.M.; Bertz, R.; Bifano, M. The pharmacokinetics of daclatasvir and asunaprevir administered in combination in studies in healthy subjects and patients infected with hepatitis C virus. *Clin Drug Investig* **2014**, *34*, 661-671, doi:10.1007/s40261-014-0219-9.
21. Duan, P.; Zhao, P.; Zhang, L. Physiologically Based Pharmacokinetic (PBPK) Modeling of Pitavastatin and Atorvastatin to Predict Drug-Drug Interactions (DDIs). *Eur. J. Drug Metab. Pharmacokinet.* **2017**, *42*, 689-705, doi:10.1007/s13318-016-0383-9.
22. Reuben, A. Chapter 31 - Hepatotoxicity of Immunosuppressive Drugs. In *Drug-Induced Liver Disease (Third Edition)*, Kaplowitz, N., DeLeve, L.D., Eds.; Academic Press: Boston, 2013; pp. 569-591.

23. Tanaka, C.; Kawai, R.; Rowland, M. Dose-dependent pharmacokinetics of cyclosporin A in rats: events in tissues. *Drug Metab Dispos* **2000**, *28*, 582-589.
24. Gertz, M.; Cartwright, C.M.; Hobbs, M.J.; Kenworthy, K.E.; Rowland, M.; Houston, J.B.; Galetin, A. Cyclosporine inhibition of hepatic and intestinal CYP3A4, uptake and efflux transporters: application of PBPK modeling in the assessment of drug-drug interaction potential. *Pharm. Res.* **2013**, *30*, 761-780, doi:10.1007/s11095-012-0918-y.
25. US Food Drug Admin. Drug Labeling-Package Insert: NEORAL(cyclosporine) capsules and oral solution [FDA application no, (NDA) 050715]. *https://www.accessdata.fda.gov/drugsatfda_docs/label/2009/050715s027,050716s028lbl.pdf [Accessed 01/03/2022]* **2009**.
26. Greenblatt, H.K.; Greenblatt, D.J. Liver injury associated with ketoconazole: review of the published evidence. *J Clin Pharmacol* **2014**, *54*, 1321-1329, doi:10.1002/jcph.400.
27. Choi, M.K.; Jin, Q.R.; Choi, Y.L.; Ahn, S.H.; Bae, M.A.; Song, I.S. Inhibitory effects of ketoconazole and rifampin on OAT1 and OATP1B1 transport activities: considerations on drug-drug interactions. *Biopharm Drug Dispos* **2011**, *32*, 175-184, doi:10.1002/bdd.749.
28. Matthew, D.; Brennan, B.; Zomorodi, K.; Houston, J.B. Disposition of azole antifungal agents. I. Nonlinearities in ketoconazole clearance and binding in rat liver. *Pharm. Res.* **1993**, *10*, 418-422, doi:10.1023/a:1018996524141.
29. Daneshmend, T.K.; Warnock, D.W.; Ene, M.D.; Johnson, E.M.; Parker, G.; Richardson, M.D.; Roberts, C.J. Multiple dose pharmacokinetics of ketoconazole and their effects on antipyrine kinetics in man. *J. Antimicrob. Chemother.* **1983**, *12*, 185-188, doi:10.1093/jac/12.2.185.
30. Marcy, T.R.; Britton, M.L.; Blevins, S.M. Second-generation thiazolidinediones and hepatotoxicity. *Ann. Pharmacother.* **2004**, *38*, 1419-1423, doi:10.1345/aph.1E072.
31. Chen, M.; Zhang, J.; Wang, Y.; Liu, Z.; Kelly, R.; Zhou, G.; Fang, H.; Borlak, J.; Tong, W. The liver toxicity knowledge base: a systems approach to a complex end point. *Clin Pharmacol Ther* **2013**, *93*, 409-412, doi:10.1038/clpt.2013.16.
32. US Food Drug Admin. Clinical Clinical Pharmacology and Biopharmaceutics Review; ACTOS (Pioglitazone Hydrochloride) [FDA application no, (NDA)021073]. **1999**.
33. Eriksson, C.; Gustavsson, A.; Kronvall, T.; Tysk, C. Hepatotoxicity by bosentan in a patient with portopulmonary hypertension: a case-report and review of the literature. *J. Gastrointestin. Liver Dis.* **2011**, *20*, 77-80.
34. US Food Drug Admin. Drug Labeling-Package Insert: (bosentan) tablets [FDA application no, (NDA) 209279]. *https://www.accessdata.fda.gov/drugsatfda_docs/label/2017/209279s000lbl.pdf [Accessed 01/03/2022]* **2017**.
35. Scotcher, D.; Jones, C.R.; Galetin, A.; Rostami-Hodjegan, A. Delineating the Role of Various Factors in Renal Disposition of Digoxin through Application of Physiologically Based Kidney Model to Renal Impairment Populations. *J Pharmacol Exp Ther* **2017**, *360*, 484-495, doi:10.1124/jpet.116.237438.
36. Scotcher, D.; Arya, V.; Yang, X.; Zhao, P.; Zhang, L.; Huang, S.M.; Rostami-Hodjegan, A.; Galetin, A. Mechanistic Models as Framework for Understanding Biomarker Disposition: Prediction of Creatinine-Drug Interactions. *CPT Pharmacometrics Syst Pharmacol* **2020**, *9*, 282-293, doi:10.1002/psp4.12508.
37. Asaumi, R.; Nunoya, K.I.; Yamaura, Y.; Taskar, K.S.; Sugiyama, Y. Robust physiologically based pharmacokinetic model of rifampicin for predicting drug-drug interactions via P-glycoprotein induction and inhibition in the intestine, liver, and kidney. *CPT Pharmacometrics Syst Pharmacol* **2022**, *11*, 919-933, doi:10.1002/psp4.12807.
38. Jamei, M.; Bajot, F.; Neuhoff, S.; Barter, Z.; Yang, J.; Rostami-Hodjegan, A.; Rowland-Yeo, K. A mechanistic framework for in vitro-in vivo extrapolation of liver membrane transporters: prediction of drug-drug interaction between rosuvastatin and cyclosporine. *Clin. Pharmacokinet.* **2014**, *53*, 73-87, doi:10.1007/s40262-013-0097-y.
39. Sun, Y.; Chothe, P.P.; Sager, J.E.; Tsao, H.; Moore, A.; Laitinen, L.; Hariparsad, N. Quantitative Prediction of CYP3A4 Induction: Impact of Measured, Free, and Intracellular Perpetrator Concentrations from Human Hepatocyte Induction Studies on Drug-Drug Interaction Predictions. *Drug Metab Dispos* **2017**, *45*, 692-705, doi:10.1124/dmd.117.075481.
40. Li, N.; Badrinarayanan, A.; Ishida, K.; Li, X.; Roberts, J.; Wang, S.; Hayashi, M.; Gupta, A. Albumin-Mediated Uptake Improves Human Clearance Prediction for Hepatic Uptake Transporter Substrates Aiding a Mechanistic In Vitro-In Vivo Extrapolation (IVIVE) Strategy in Discovery Research. *AAPS J* **2020**, *23*, 1, doi:10.1208/s12248-020-00528-y.
41. Riede, J.; Camenisch, G.; Huwyler, J.; Poller, B. Current In Vitro Methods to Determine Hepatic Kp(uu): A Comparison of Their Usefulness and Limitations. *J. Pharm. Sci.* **2017**, *106*, 2805-2814, doi:10.1016/j.xphs.2017.03.025.
42. Cremer, J.E.; Seville, M.P. Regional brain blood flow, blood volume, and haematocrit values in the adult rat. *J. Cereb. Blood Flow Metab.* **1983**, *3*, 254-256, doi:10.1038/jcbfm.1983.35.
43. Lee, H.B.; Blaufox, M.D. Blood volume in the rat. *J. Nucl. Med.* **1985**, *26*, 72-76.
44. Jobin, J.; Bonjour, J. Measurement of glomerular filtration rate in conscious unrestrained rats with inulin infused by implanted osmotic pumps. *Am. J. Physiol. Renal Physiol.* **1985**, *248*, F734-F738.
45. US Food Drug Admin. Drug Labeling-Package Insert: EOVIST (Gadoxetate Disodium) Injection [FDA application no, (NDA) 022090]. *https://www.accessdata.fda.gov/drugsatfda_docs/label/2018/022090s016lbl.pdf [Accessed 5/4/2019]* **2018**.

Disclaimer/Publisher's Note: The statements, opinions and data contained in all publications are solely those of the individual author(s) and contributor(s) and not of MDPI and/or the editor(s). MDPI and/or the editor(s) disclaim responsibility for any injury to people or property resulting from any ideas, methods, instructions or products referred to in the content.
